# Supplementary material for: A neuronal basis of iconic memory in macaque primary visual cortex
Source: Curr Biol. 2021 Dec 20;31(24):5401–5414.e4. doi: 10.1016/j.cub.2021.09.052 (PMC8699744; doi:10.1016/j.cub.2021.09.052)
Supplement: Document S1. Figures S1–S4 [file mmc1.pdf]

Current Biology, Volume *31*

Supplemental Information

A neuronal basis of iconic memory

in macaque primary visual cortex

Rob R.M. Teeuwen, Catherine Wacongne, Ulf H. Schnabel, Matthew W. Self, and Pieter R. Roelfsema

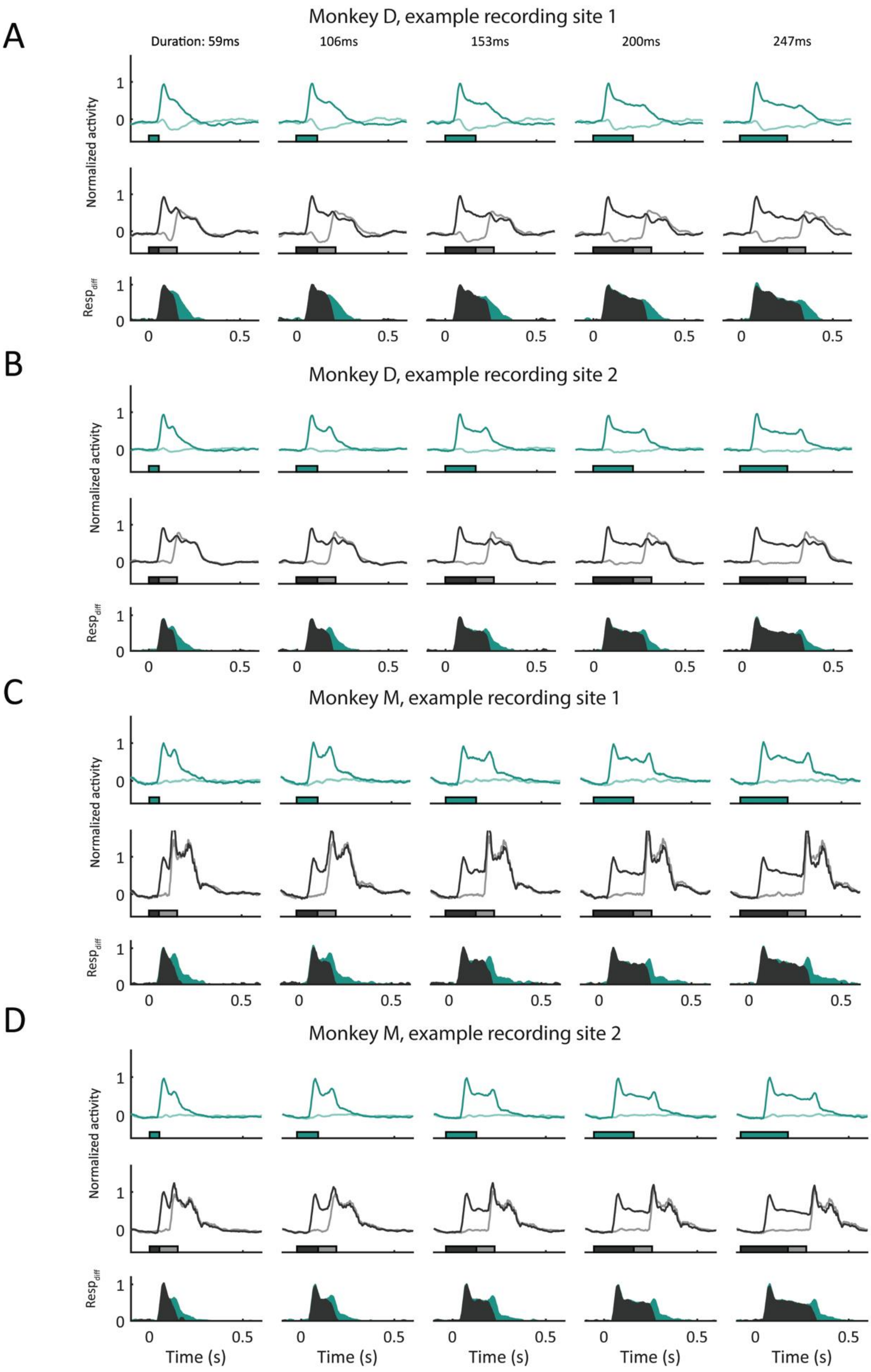
A
Monkey D, example recording site 1
Duration: 59ms
106ms
153ms
200ms
247ms
Normalized activity
$Resp_{diff}$
B
Monkey D, example recording site 2
C
Monkey M, example recording site 1
D
Monkey M, example recording site 2
Time (s)

Figure S1. The neuronal worth at example V1 recording sites. Related to Figure 3. (A,B) Representative V1 recording sites in the worth experiment in monkey D. Dark green and black lines, activity elicited by the stimulus with the discriminant segment in the RF. Light green and grey lines, activity elicited by the stimulus with the discriminant segment outside the RF. In some trials we presented a mask (black, grey) and in other trials the mask was absent (dark and light green). Bars on the x-axis indicate stimulus and mask duration. The panels below represent $Resp_{diff}$, which is the difference in activity elicited by the conditions with the discriminant segment inside and outside the RF in the presence (grey area) and absence of masking (green area). The extra activity difference that occurs without masking is shown as a green area and corresponds to our measure of the neuronal worth. (C,D) Representative recording sites in monkey M.

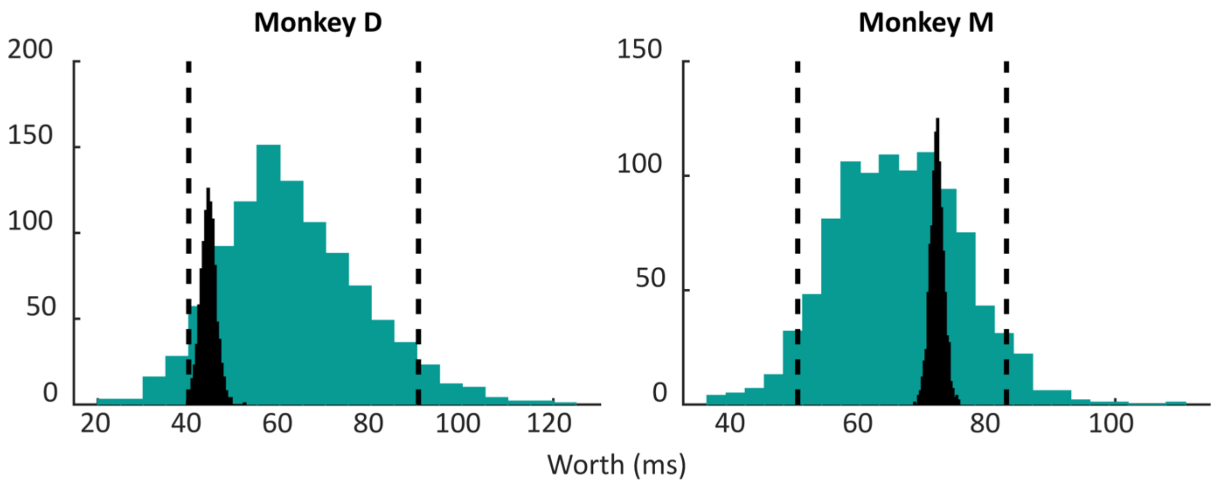

Figure S2. Confidence intervals of the behavioral and neuronal worth. Related to Figure 3. The distributions obtained with bootstrapping for our estimates of behavioral worth (green) and neuronal worth (black). The dashed lines represent the 95% confidence interval of the behavioral worth. The distributions are overlapping, which indicates that the neuronal worth is compatible with the behavioral worth.

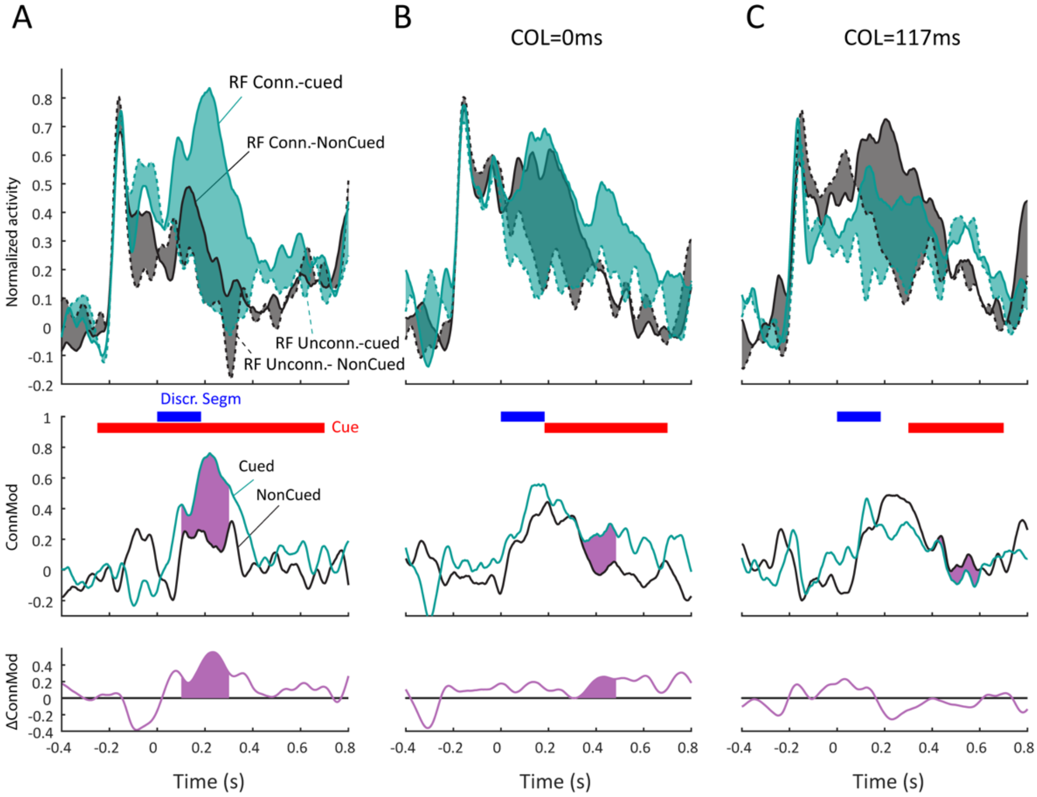

Figure S3. Interactions between iconic memory and the central cue in monkey D. Related to Figure 7. (A-C) V1 activity in the pre-cue condition (A), COL=0 condition (B), and COL=117ms (C), compare to Figure 7C-E (data of monkey M). The contour element in the RF was either connected to the discriminant segment (continuous traces) or not (dashed traces). The pair of curves covering the RF could be cued (green) or not (black). Responses were stronger if the contour in the RF was connected to the discriminant segment than if it was not. ConnMod is the extra activity (green and grey areas) elicited if the RF-contour is connected. The middle panels show the time-course of the connectedness modulation for the cued and non-cued pair of curves, and ΔConnMod (purple area). The lower panels show the time-course of the cueing effect.

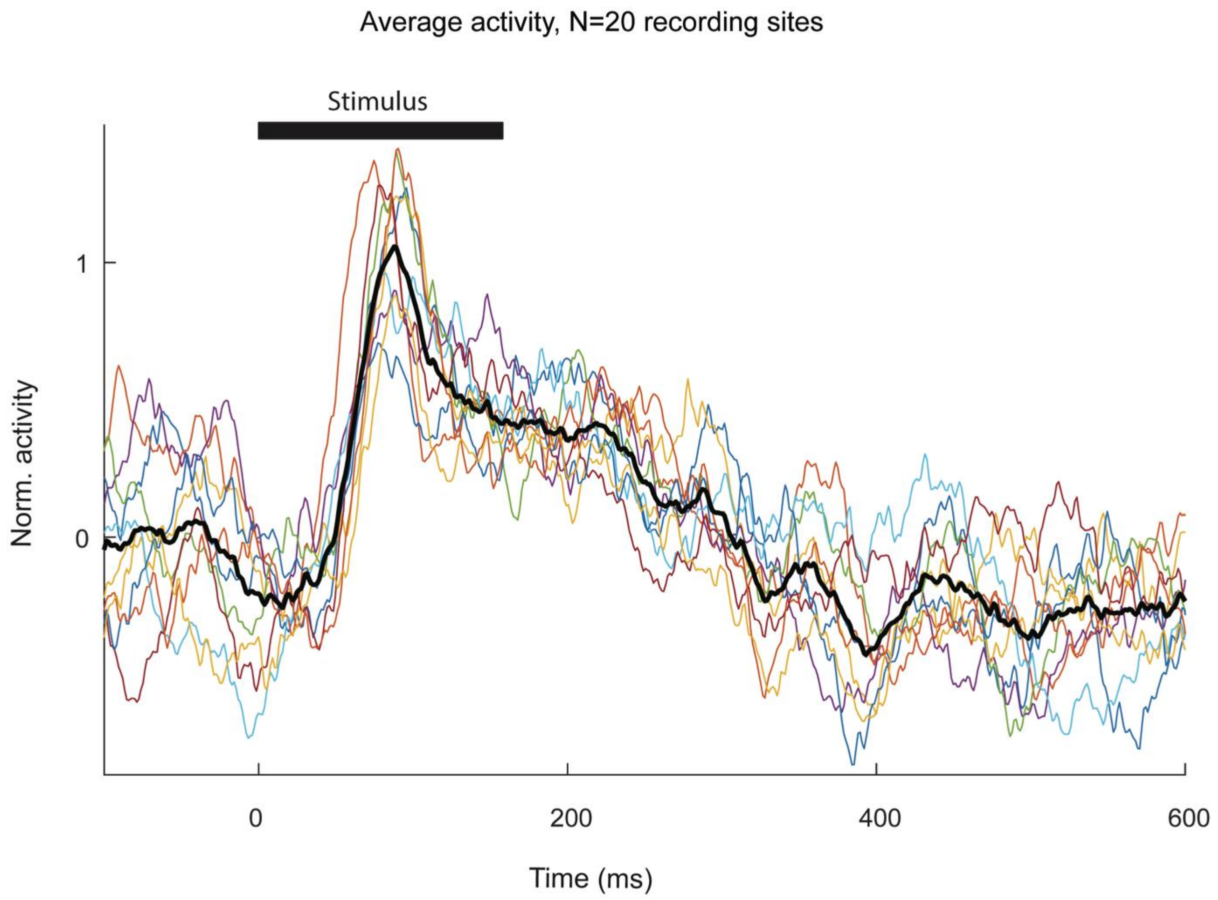

Figure S4. Activity on single trials. Related to Figure 5. Average V1 activity on a number of successive trials averaged across 20 recording sites in monkey D (stimulus duration 158ms, no mask). Colored lines, individual trials. Black line, average across trials in this condition.
