# Supplementary material for: A neuronal basis of iconic memory in macaque primary visual cortex
Source: Curr Biol. 2021 Dec 20;31(24):5401–5414.e4. doi: 10.1016/j.cub.2021.09.052 (PMC8699744; doi:10.1016/j.cub.2021.09.052)
Supplement: Document S2. Article plus supplemental information [file mmc3.pdf]

Current Biology

Article

A neuronal basis of iconic memory in macaque primary visual cortex

Graphical abstract

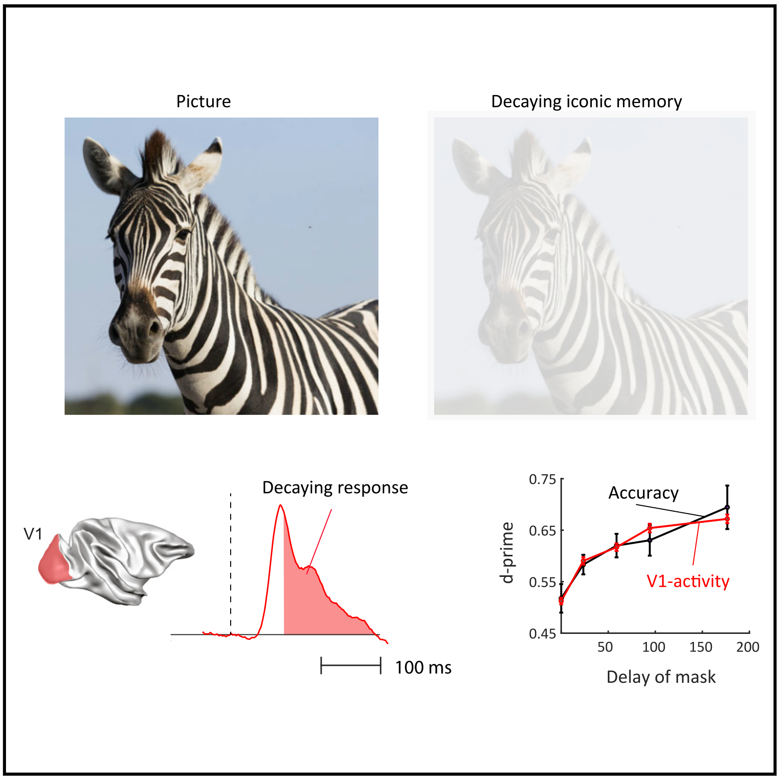

Authors

Rob R.M. Teeuwen,
Catherine Wacongne, Ulf H. Schnabel,
Matthew W. Self, Pieter R. Roelfsema

Correspondence

p.roelfsema@nin.knaw.nl

In brief

Teeuwen et al. examine the neuronal mechanisms for iconic memory, the short-lasting, detailed representation of a briefly presented stimulus. They demonstrate that the decaying response of neurons in primary visual cortex (V1) predicts the accuracy of iconic memory and show how iconic memories can be grabbed by attention and abolished by masking.

Highlights

- Iconic memory is a short-lasting, detailed representation of a brief visual image
- Decaying V1 activity after a briefly presented stimulus accounts for iconic memory
- Masks abolish iconic memory and erase the V1 representation of an image
- Attention interacts with iconic memory to boost relevant V1 activity

Teeuwen et al., 2021, Current Biology *31*, 5401–5414
December 20, 2021 © 2021 The Authors. Published by Elsevier Inc.
https://doi.org/10.1016/j.cub.2021.09.052

CellPress

Current Biology

CellPress
OPEN ACCESS

Article

A neuronal basis of iconic memory in macaque primary visual cortex

Rob R.M. Teeuwen,[1] Catherine Wacongne,[1] Ulf H. Schnabel,[1] Matthew W. Self,[1] and Pieter R. Roelfsema[1,2,3,4,5,*]
[1]Department of Vision & Cognition, Netherlands Institute for Neuroscience, Meibergdreef 47, 1105 Amsterdam, the Netherlands
[2]Psychiatry Department, Academic Medical Center, University of Amsterdam, Nieuwe Achtergracht 129-B, 1018 Amsterdam, the Netherlands
[3]Department of Integrative Neurophysiology, Center for Neurogenomics and Cognitive Research, VU University, De Boelelaan 1085, 1081 Amsterdam, the Netherlands
[4]Twitter: @Pieters_Tweet
[5]Lead contact
*Correspondence: p.roelfsema@nin.knaw.nl
https://doi.org/10.1016/j.cub.2021.09.052

SUMMARY

After a briefly presented visual stimulus disappears, observers retain a detailed representation of this stimulus for a short period of time. This sensory storage is called iconic memory. We measured iconic memory in the perception of monkeys and its neuronal correlates in the primary visual cortex (area V1). We determined how many milliseconds extra viewing time iconic memory is worth and how it decays by varying the duration of a brief stimulus and the timing of a mask. The V1 activity that persists after the disappearance of a stimulus predicted accuracy, with a time course resembling the worth and decay of iconic memory. Finally, we examined how iconic memory interacts with attention. A cue presented after the stimulus disappears boosts attentional influences pertaining to a relevant part of the stimulus but only if it appears before iconic memory decayed. Our results relate iconic memory to neuronal activity in early visual cortex.

INTRODUCTION

Our visual system takes in a large amount of information with every fixation of the eyes. Only a fraction of it can be processed and remembered. There are different forms of memory that act on distinct timescales, including working memory, which is associated with persistent activity of neurons,[1,2] and long-term memory, which is laid down in the patterns of synaptic weights. In visual perception, there also exists a short-lasting, very-high-capacity memory store that is known as iconic memory.[3–7] Sperling[3] conducted seminal studies on this form of memory. In one of his studies, subjects saw a display with 12 letters and digits ordered in three rows during a 50-ms time interval (Figure 1A). In one condition, called "whole report," the subjects were asked to report all letters that they remembered. They were able to report four letters, on average, a number that hardly increased for longer stimulus durations, up to 500 ms. This result implies that the capacity limitation was not caused by the limited visibility of the briefly presented letters but by an inability to memorize more than four items, a finding which was reproduced many times.[8–12] According to contemporary terminology, the four items are in working memory, which is a memory store that can last for seconds and is relatively resilient against interference by subsequent visual stimuli.

Sperling[3] also carried out a "partial report" version of the experiment, which revealed compelling evidence for another storage mechanism that precedes working memory. In this version of the task, the subjects heard a tone immediately after stimulus offset, cueing subjects to report a specific row of letters (Figure 1A). The remarkable finding was that subjects were typically able to reproduce all letters of a cued row. Apparently, they had access to a representation of all 12 letters, even though they were not visible anymore, and they could report the relevant subset upon cueing. The inferred early, high-capacity buffer that contains all letters was later called "iconic memory." Iconic memory was found to decay quickly because a delay of a few hundred milliseconds between stimulus offset and cue onset decreased the subject's capacity to report the letters to the level without cueing. Apparently, iconic memories provide a rich representation of the visual scene but are short lived. Additional research demonstrated that iconic memories are erased when they are overwritten by another visual stimulus or mask.[4,5,13]

Although previous studies described the properties of iconic memory at a perceptual level, we know remarkably little about its neuronal mechanisms and the readout of this high-capacity memory buffer. A briefly presented visual stimulus elicits a response that is selective for the stimulus and outlasts the stimulus duration in the primary visual cortex (area V1) and higher visual cortical areas, including the inferotemporal cortex.[14–18] Furthermore, in all these areas, the delayed stimulus-selective responses can be interrupted by a mask.[15,16,19,20] A modeling study therefore proposed that the late, decaying phase of the neuronal response elicited by a brief visual stimulus might correspond to the iconic memory trace.[21] This hypothesis has, to our knowledge, not yet been tested experimentally. In the present study, we aimed to assess the relation between iconic memory and the late, decaying phase of V1 activity elicited by brief stimuli. We assessed two measures of iconic memory in the behavior of macaque monkeys, "worth" and "decay," and compared

This is an open access article under the CC BY license (http://creativecommons.org/licenses/by/4.0/).

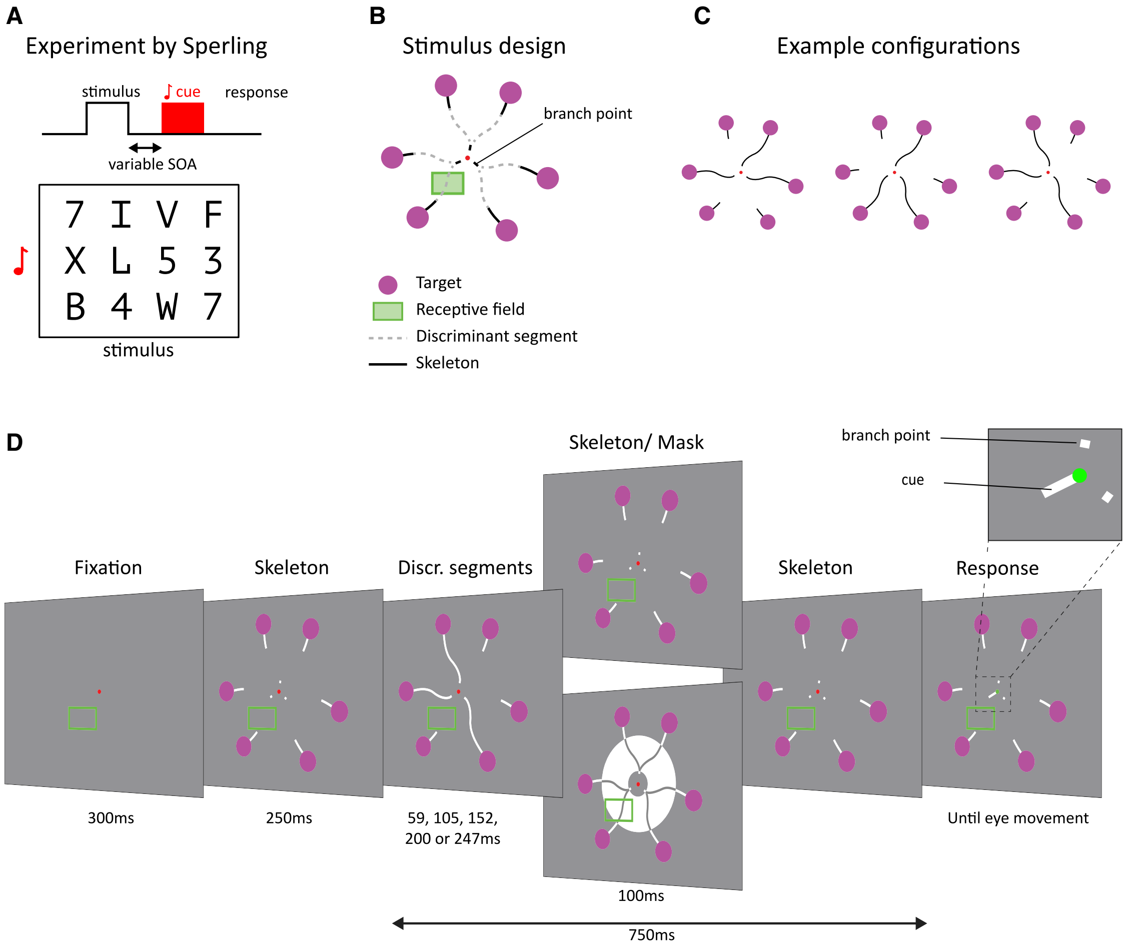

Figure 1. Iconic memory paradigm

(A) Sperling's experiment. The subjects saw a display with 12 letters for a short amount of time. In the partial report version of the paradigm, an auditory cue indicated which row had to be reported.

(B) Stimulus design. The monkeys made eye movements to one of six purple targets, organized in pairs. The actual stimulus consisted of white curves on a gray background (as in D). The contour elements shown as black solid lines are always visible (skeleton), and a subset of the gray dashed contour elements (discriminant segments) are only displayed briefly. In this example stimulus, the RF (green rectangle) fell on one of the discriminant segments.

(C) Example configurations. On each trial, one of the discriminant segments is shown per pair so that only one of the purple circles of that pair is connected to the branch point.

(D) Stimulus sequence. After the monkey had maintained gaze at the fixation point for 300 ms, the skeleton was visible for 250 ms. Next, the three discriminant segments were shown for a variable duration (58, 105, 152, 200, or 247 ms). After the discriminant segments disappeared, a meta-contrast mask was displayed for 100 ms on half of the trials, whereas the skeleton was displayed in the other half of the trials. At a delay of 750 ms after the onset of the discriminant segments, a central cue appeared (see inset), probing one of the pairs. At this moment, the fixation point became green, cueing the monkey to make an eye movement to the purple circle that had been connected to the branch point of the cued pair. The green rectangle denotes the RF of the recorded neurons and was not visible to the monkey.

these behavioral measures to properties of the late phase of the response of V1 neurons.

The worth quantifies the amount of information contained in iconic memory, in terms of extra stimulus viewing time, i.e., how much extra viewing time the icon is worth. Loftus and colleagues[22] described a straightforward method to measure the worth. They compared the accuracy of observers in a condition in which a stimulus was followed by a mask overwriting iconic memory to that in a condition without a mask. The worth is the extra time for which a masked stimulus needs to be visible before it can be recalled with the same accuracy as an unmasked stimulus. They observed that worth of iconic memory in human observers varies between 70 and 100 ms. Loftus et al.[22] also measured the shape of the decay of iconic memory by varying the delay between the offset of the stimulus and the onset of the mask. They observed that iconic memory decayed with a time constant of around 100 ms, although studies using other procedures observed longer time constants, up to 400 ms.[23]

In the present study, we measured the worth and decay of iconic memory and compared it to the activity of neurons in area V1 using a modified curve-tracing task (Figures 1B–1D).[24] The monkeys directed their gaze to a fixation point and had to determine which of six purple circles was connected to this fixation point by a curve that consisted of a number of contour

CellPress
OPEN ACCESS

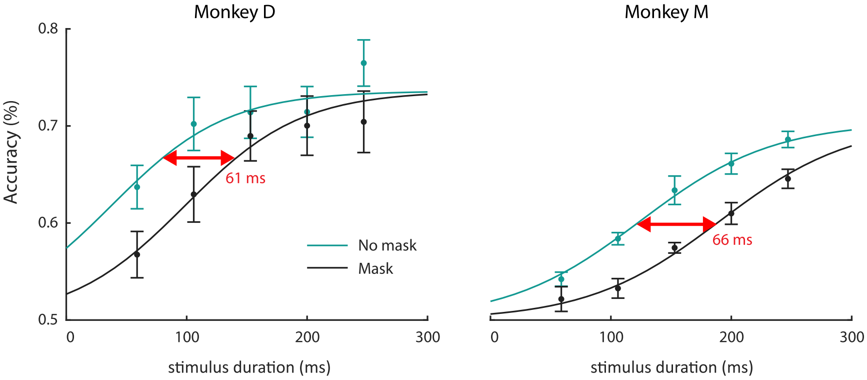

Figure 2. Behaviorally determined worth
The monkeys' accuracy (y axis) in the presence (black traces) and absence of a mask (green traces) depended on the duration for which the discriminant segments were visible. The lines represent fits of sigmoidal functions, and the horizontal shift (red arrow) represents the worth. Error bars, SEM across sessions.

elements that were presented in a piecemeal manner. There were three pairs of curves, each starting at a branch point close to the fixation point (Figure 1B). On every trial, a ''discriminant'' contour element appeared for only one of the curves in each pair, connecting the branch to one of the purple circles (Figures 1C and 1D; Video S1). The monkey had to memorize the three discriminant contour elements. After a variable delay after the discriminant segment disappeared, we presented a central cue, which was a small contour element connecting the fixation point to one of the three branch points. The monkeys reported which of the two purple circles had been connected by a curve to the respective branch point with an eye movement. To examine the role of iconic memory, we presented a meta-contrast mask in 50% of trials to erase iconic memory for the discriminant segments (Figure 1D).

It is of interest to compare this design to that by Sperling.[3] Instead of letters, the monkeys had to memorize discriminant contour elements, and the later central cue played the same role as the auditory cue in Sperling's task. We examined the behavioral worth by comparing the accuracy on masked and non-masked trials and measured a neuronal worth by monitoring the neuronal activity elicited by the discriminant segments in area V1. In addition, we varied the delay between stimulus offset and mask onset to assess the decay of iconic memory. We found that the neuronal measures for worth and decay were in the same range as the behavioral measures and that variability in the strength of the decaying V1 response after the stimulus is no longer visible predicted the monkeys' accuracy, implying it provides a neuronal correlate of iconic memory. We also reproduced the attentional cueing effect at the behavioral and neuronal level. It was most pronounced for cues that appeared before or shortly after the presentation of the discriminant contour elements, before iconic memory decayed.

RESULTS

To examine the relation between iconic memory and neuronal activity in V1, we first examined the worth and compared it to V1 activity in the presence and absence of a mask. The worth of iconic memory is measured as extra viewing time in milliseconds, e.g., the worth would be 100 ms if a masked stimulus would need to be presented 100 ms longer to compensate for the loss of accuracy caused by the mask.

Behavioral worth of iconic memory

In the curve-tracing task, three pairs of two purple circles could be briefly connected to three branch points by discriminant contour elements, and one of these branch points was later connected to the fixation point by a cue (Figures 1B–1D). We presented the discriminant contour elements for different durations, and the monkeys had to memorize the three segments because the cue, i.e., the connection to the fixation point, was presented after the discriminant segments disappeared, at the end of the trial. We expected that iconic memory would be beneficial in this task, because the discriminant segments were displayed only briefly. To measure the performance in the different conditions, we collected a total of 15,133 trials (in 13 sessions) and 20,891 trials (7 sessions) in monkey D and monkey M, respectively.

In half of the trials, we erased iconic memory with a meta-contrast mask with a duration of 100 ms (Figure 1D). Masking decreased the accuracy of both monkeys (likelihood ratio test, testing whether accuracy depended on presence of the mask; see STAR Methods, $p = 3.2 \cdot 10^{-10}$ for monkey D and $p = 1.5 \cdot 10^{-11}$ for monkey M; Figure 2). The effect of the mask was well described as a rightward shift of the psychometric function. To estimate the worth, we fitted a sigmoidal function to the accuracy in both conditions and measured the horizontal shift (Figure 2). In monkey D, the estimated worth was 61 ms with a 95% confidence interval of [39 ms, 91 ms] (estimated using a bootstrapping procedure; see STAR Methods). The behavioral worth estimate for monkey M was 66 ms with a 95% confidence interval of [47 ms, 86 ms]. Hence, iconic memory appears to add just over 60 ms to the effective presentation time of a briefly presented stimulus.

The accuracy of the monkeys asymptoted at a value below 100%. This asymptote provides a measure of the maximum number of items that the monkeys held in working memory. An accuracy of 100% would imply that they held all three discriminant segments in memory and an accuracy of 50% (chance level) that they remembered none. The asymptotic number of items in working memory can be approximated as $6 \times (\text{accuracy} - 0.5)$, and it was 1.42 for monkey D (asymptotic accuracy, 73.6% ± 1.5%; mean ± SEM) and 1.22 for monkey M (asymptotic accuracy, 70.4% ± 2.1%).

Neuronal worth can account for behavioral worth

To estimate the neuronal worth, we recorded the activity of V1 neurons in both monkeys during the task using chronically implanted electrode arrays (Utah probes). We recorded the activity of 20 V1 recording sites in monkey D and 18 recording sites in monkey M during the same sessions that were used to compute

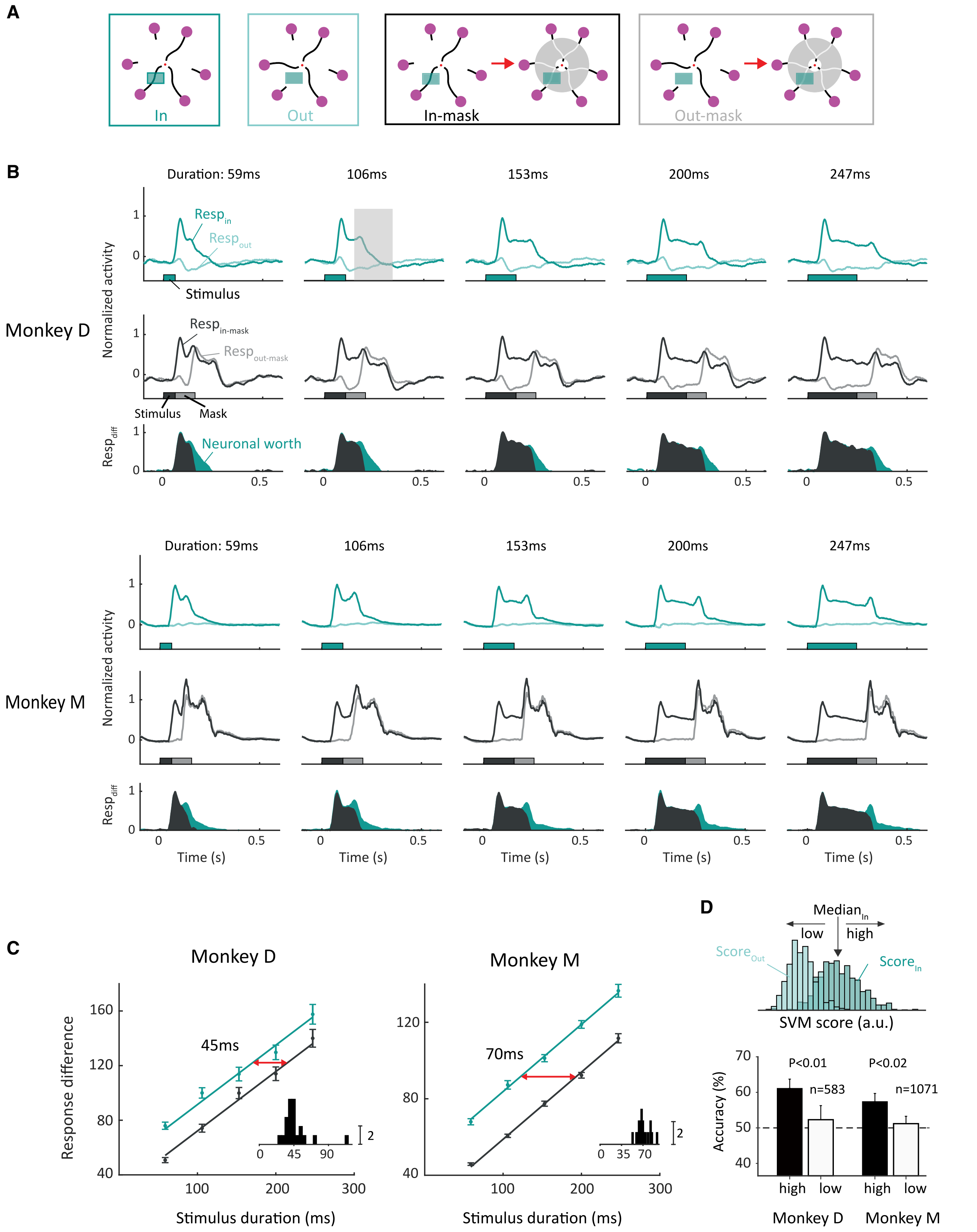

Figure 3. The neuronal worth in V1

(A and B) Activity elicited by the stimulus with the discriminant segment in the RF (dark green, black) or not in the RF (light green, gray) in the presence (black, gray) and absence of a mask (dark and light green) in monkey D (A) and monkey M (B). The insets above illustrate the different stimulus conditions. The panels below

(legend continued on next page)

the behavioral worth. We ensured that the receptive fields (RFs) of the V1 neurons fell on one of the discriminant contour elements and not on other parts of the stimulus.

As expected, the appearance of the discriminant segment in the V1 RF elicited a visually driven response (dark green traces in Figures 3A and 3B), $Resp_{In}$, with a duration that depended on presentation time. The disappearance of the discriminant segment elicited a second small peak in the response of some V1 neurons, and thereafter, the activity gradually decreased to the level of spontaneous activity. The other discriminant segment of the same curve pair did not elicit a V1 response (light green in Figures 3A and 3B), $Resp_{Out}$, because it did not fall in the RF. As a measure for the information that could be relayed by the V1 neurons about the discriminant segment at each moment, we calculated the response difference, $Resp_{Diff} = Resp_{In} - Resp_{Out}$ (Figures 3A and 3B, green area in the lower panels), which evidently increased for longer stimulus durations (green line in Figures 3C and 3D).

In trials with the meta-contrast mask, the onset of the mask caused a sharp increase in neural activity (black and gray traces in Figures 3A and 3B), irrespective of the location of the discriminant segment. Accordingly, the mask quickly abolished $Resp_{Diff}$ and curtailed the information about the location of the discriminant segment (compare gray and green areas in lower panels of Figures 3A and 3B). Neurons at individual recording sites exhibited the same effect (see Figure S1 for representative recording sites from both monkeys). To assess significance, we fit a general linear model to the area of $Resp_{Diff}$ across recording sites with a single slope term for stimulus duration (resulting in parallel lines for both levels of masking; Figures 3C and 3D). Both the main effect of stimulus duration and the main effect of masking were highly significant in both monkeys (monkey D: $t_{duration} = 41.4$, $p_{duration} = 2.7 \cdot 10^{-93}$, $t_{mask} = -14.0$, $p_{mask} = 1.9 \cdot 10^{-30}$; monkey M: $t_{duration} = 73.0$, $p_{duration} = 9.8 \cdot 10^{-125}$, $t_{mask} = -38.6$, $p_{mask} = 6.5 \cdot 10^{-83}$). The horizontal shift between the two lines (Figures 3C and 3D) represents the neuronal worth, which was 45 ms for monkey D (95% confidence interval [41 ms, 50 ms]) and 70 ms for monkey M (95% confidence interval [65 ms, 72 ms]). The neuronal worth fell well within the confidence intervals of the behavioral worth of the two monkeys, and it is therefore compatible with the behavioral worth estimate (Figure S2). The behavioral worth estimates in the two monkeys (61 and 66 ms) were more similar than the neural worth estimates (45 and 70 ms). However, our design compares conditions within monkeys, and it is not suitable for an analysis of individual differences, which would require testing many more monkeys.

The strength of decaying V1 activity after stimulus offset predicts accuracy

Although a statistical comparison across monkeys was not feasible, we were able to investigate whether variations in the level of decaying activity in V1 across trials predicts the accuracy of the two monkeys. We used a support vector machine (SVM) to achieve maximal discriminative power across V1 recording sites, training it to discriminate between trials with the discriminant segment inside and outside of the RF, based on the decaying response phase (50–250 ms after stimulus offset; gray shaded region in Figure 3B). The SVM defines a hyperplane that maximally separates trials in which the discriminant segment had fallen into the RF from those in which it had not, and the distance from this hyperplane can be quantified with an SVM score. The SVM score is high on trials in which V1 neurons had been activated by the discriminant segment and low on trials where it fell outside the RF ($Score_{In}$ versus $Score_{Out}$ in Figure 3D). To investigate whether variations in the magnitude of the decaying response phase are related to accuracy, we used the SVM score to sort trials in which the discriminant segment had been in the RF ($Score_{In}$) into two groups, with high and low levels of decaying activity (median split), using stimulus durations of 59, 106, and 153 ms (accuracy asymptoted for longer durations, leaving less room for it to be influenced; Figure 2). We first investigated the monkeys' accuracy on trials in which the central cue instructed them to report about the RF stimulus. The monkeys' performance was better if the decaying response was strong than if it was weak (monkey D, 61% versus 52%; monkey M, 57% versus 51%; Figure 3D). These accuracy differences were significant (monkey D, $p < 0.01$; monkey M, $p < 0.02$; bootstrap test). We repeated the analysis for trials in which the discriminant segment fell in the RF, but the central cue instructed the monkeys to report about another pair of curves, not in the RF. On these trials, the decaying activity level did not predict accuracy (the other two cue locations and two monkeys; all $p > 0.05$; bootstrap test). We conclude that variability in the strength in the V1 decaying response phase predicts accuracy but only when the monkeys report about the RF stimulus.

The decay of neuronal worth accounts for the decay of behavioral worth

The worth provides an estimate of the effective extra viewing time that iconic memory is equivalent to. We next examined the time course of iconic memory. Loftus et al.[22] showed how to estimate iconic memory's decay by assessing the

represent $Resp_{diff}$, which is the difference in activity elicited by the conditions with the discriminant segment inside and outside the RF in the presence (gray area) and absence of masking (green area). The extra activity difference that occurs without masking is shown as a green area and corresponds to our measure of the neuronal worth.

(C) Integrated $Resp_{diff}$ as function of the stimulus duration in the presence (black) and absence of a mask (green). The data points correspond to the shaded areas in the bottom row of (A) and (B). Lines represent linear fits and error bars the SEM across recording sites. The horizontal shift between the lines (red arrow) corresponds to the neuronal worth. Insets, distribution of the worth estimates determined for individual recording sites.

(D) Upper: SVM that maximally separates trials with the discriminant segment inside and outside the RF of the V1 recording sites in monkey D. Histograms, distribution of SVM scores across trials with the discriminant segment inside (dark green) and outside the RF (light green). We used a time window from 50 to 250 ms after stimulus offset (gray rectangle in B) on trials with shorter stimuli ($\leq$153 ms). We performed a median split on the SVM score on trials with decaying activity elicited by the discriminant segment to compare the monkeys' accuracy on trials with high and low V1 activity. We here selected trials in which the monkey was cued to report about the pair of curves in the V1 RFs (n = 1/3 of these trials). The accuracy was higher on trials with a higher level of decaying V1 activity (black bars) than if the V1 activity was low (white bars). n, number of trials in the analysis. The p-values indicate the result of a bootstrap test.

See also Figures S1 and S2.

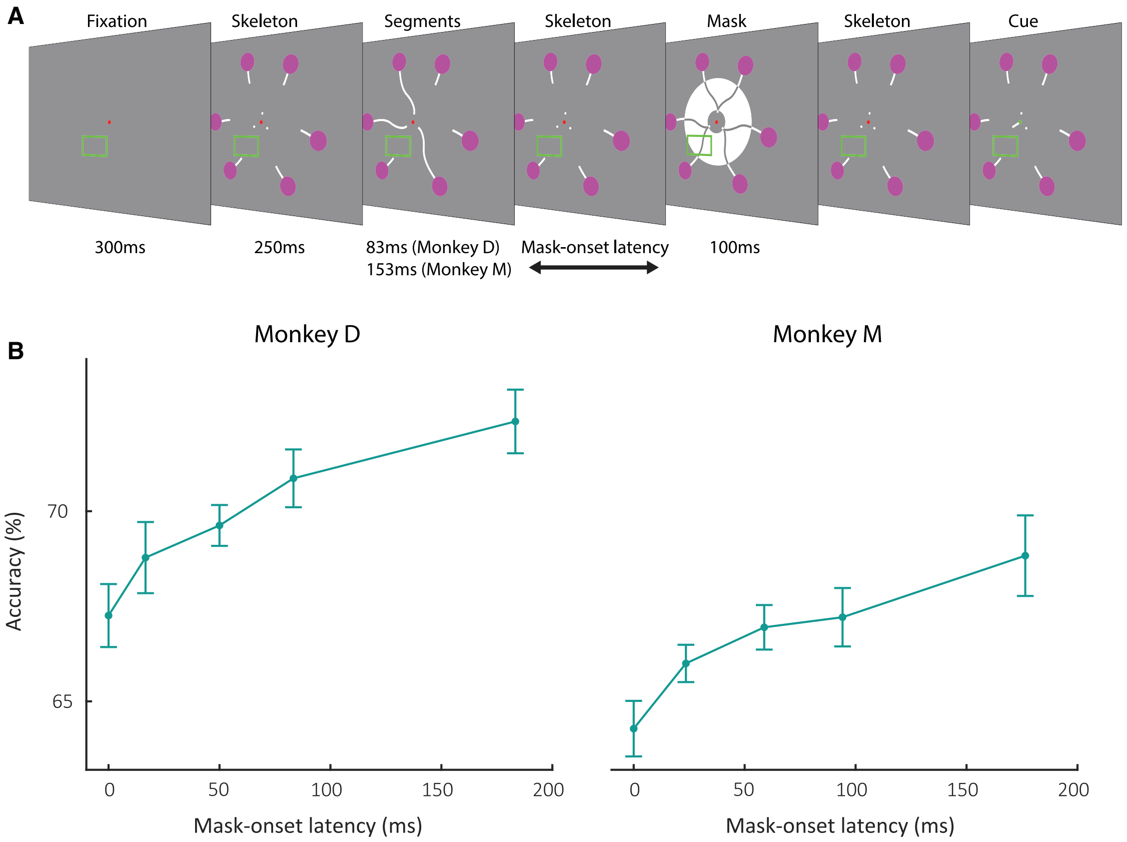

Figure 4. Decay of iconic memory

(A) The discriminant segments were shown for a constant duration, and the delay between their offset and the onset of the mask (MOL, mask onset latency) was varied.

(B) Accuracy of monkey D (left) and M (right) as function of MOL. Error bars, SEM across sessions.

accuracy for different delays between stimulus offset and mask onset. If the mask is not presented immediately after the offset of the stimulus but delayed by a mask onset latency (MOL), the increase in accuracy gives insight in the information available in iconic memory during the MOL, i.e., the interval's partial worth.

The monkeys performed a version of the task in which the stimulus duration stayed the same, and only the MOL varied across trials (Figure 4A). We recorded 10,778 trials (10 sessions) in monkey D and 21,522 trials in monkey (9 sessions). For monkey D, the presentation time of the discriminant segments was 83 ms. Monkey M had a lower accuracy, and we therefore presented the discriminant segments for 153 ms. The accuracy of both monkeys was lowest at a MOL of 0, because the mask completely blocked iconic memory. Performance increased for longer MOLs because less of the icon was blocked by the mask (Figure 4B; likelihood ratio test; $p = 5.4 \cdot 10^{-3}$ for monkey D and $p = 3.4 \cdot 10^{-4}$ for monkey M). The accuracy increase was steeper at short MOLs and decreased at longer values. The diminishing returns at longer intervals gives insight into the decay of iconic memory. The monkeys' accuracy depends on the total information provided by iconic memory up to the MOL, in other words, the masking curve represents the integral of information provided by iconic memory. Vice versa, the temporal profile of the iconic memory trace should correspond to the masking curve's derivative.[22]

We next examined how the simultaneously recorded late V1 activity depends on the MOL, assessing the partial worth at different time points (Figure 5) for 20 recording sites in monkey D and 18 recording sites in monkey M. One of the discriminant segments fell in the neurons' RF and elicited a visual response, $Resp_{In}$ (green trace in Figures 5B and 5C), whereas the other segment did not ($Resp_{Out}$, black trace). When the MOL is 0 ms, iconic memory is prevented by the mask so that the difference in activity, $Resp_{Diff}$, is driven by the visual stimulus only (gray area in the lower panels of Figures 5B and 5C). $Resp_{Diff}$ increased monotonically with the MOL, due to an increasing contribution of the late V1 response when the mask was delayed (light green area). As in the previous experiment, the integral of $Resp_{Diff}$ (dark plus light green area in Figures 5B and 5C) provides an estimate of the V1 information about the location of the discriminant segment, and it increased for larger values of the MOL (Figures 5B–5D).

How well does the neuronal decay function account for the behavioral decay function? To address this question, we compared neuronal activity in V1 to the temporal profile of the behavioral iconic memory decay function. Specifically, we first determined the behavioral d-prime (derived from the data in Figure 4B) as function of the MOL because, unlike d-prime,

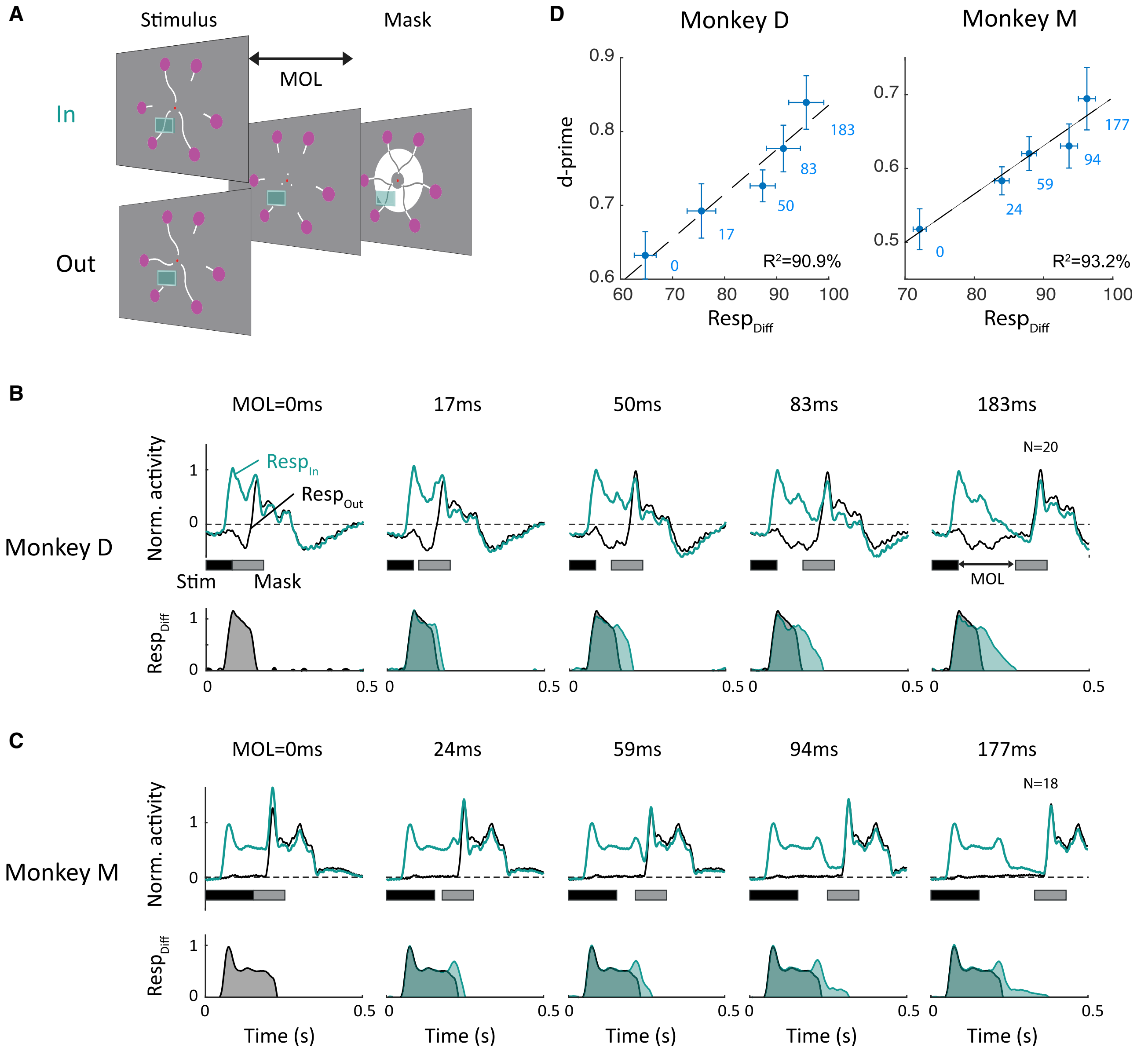

Figure 5. Iconic decay function in V1

(A) The discriminant segment appeared either inside or outside the RF, and the mask appeared after the MOL.

(B and C) Average V1 activity elicited in the condition with the discriminant segment inside (green) or outside the RF for each MOL for monkey D (B) and M (C). $Resp_{Diff}$ represents the activity difference and increases for larger values of the MOL. The visually driven response can be estimated from the MOL = 0 condition (gray area), and the extra activity elicited at longer MOLs represents a neuronal estimate of the partial worth of iconic memory (green areas).

(D) Correlation between the extra neuronal information ($Resp_{Diff}$ in C) and behavioral d-prime across the different mask-onset latencies. Horizontal error bars, SEM of $Resp_{Diff}$ across recording sites, estimated as the area under the curve in (B) and (C). Vertical error bars, SEM of d-prime across recording sessions. See also Figure S4.

increases in accuracy with improved visibility are smaller if it is already close to 100% (a "ceiling effect"). We then examined how well $Resp_{Diff}$ accounted for the variance in d-prime, using linear regression (Figure 5D). $Resp_{Diff}$ explained 90.9% (p = 0.012) of the variability in d-prime in monkey D and 93.2% (p = 0.0077) in monkey M. The correlation between the neuronal and behavioral decay functions provides further support for the hypothesis that the late V1 response after stimulus offset provides a neuronal correlate of iconic memory.

Partial report

In the partial report version of Sperling's experiment,[3] the subjects heard an auditory tone that indicated which items needed to be reported (Figure 1A). Later work replicated the results with a visual cue.[4,7] Only if the cue appeared during the phase in which the items were still in iconic memory, subjects could selectively report the cued items, whereas they did not benefit if the cue was presented at a later time point, when iconic memory had decayed.

We created a partial report version of our paradigm by varying the delay between the central cue and the discriminant segments (Figure 6A). In this version, we did not use masking so that the monkeys could use the full iconic memory trace of the discriminant segments, which were shown for 183 ms in monkey D and 294 ms in monkey M. In one condition (pre-cue), we presented the central cue before the onset of the discriminant segments so that the animal could focus on the relevant curve pair, which was expected to result in a high accuracy. In the other conditions, the cue was presented after the offset of the discriminant segments with a cue-onset latency (COL) of 0, 47, 117, or 176 ms for monkey M and a COL of 0, 33, 67, or 117 ms for monkey D (Figure 6A). Trials with different COLs were randomly interleaved. We expected a maximal benefit from iconic memory for short values of the COL and poorer performance at larger values, when iconic memory had decayed. Figure 6B shows that the behavioral results in this paradigm are in line with the original findings by Sperling.[3] As expected, the accuracy was higher for the condition in which the cue was presented throughout the trial (pre-cue) than the condition in which the cue was presented immediately after the offset of the discriminant segments (paired t test across sessions; $p = 6.5 \cdot 10^{-3}$, $t = 3.2$, $df = 15$ for monkey D and $p = 1.9 \cdot 10^{-8}$, $t = 22.0$, $df = 8$ for monkey M). Importantly, the accuracy for the immediate cue (COL = 0) was, in turn, higher than that for the longest COL (117 ms in monkey D and 176 ms in monkey M; $p = 6.1 \cdot 10^{-4}$, $t = 4.3$, $df = 15$ for monkey D and $p = 1.5 \cdot 10^{-5}$, $t = 9.3$, $df = 8$ for monkey M). The early cue apparently allowed the monkeys to take advantage of the iconic memory trace of the discriminant segment of the cued pair of curves.

We know from previous work that human observers who trace a target curve among distractor curves direct their attention to all contour elements of the target curve.[25] In monkeys, it is possible to monitor this attentional selection process by recording neuronal activity in V1, because the activity that is elicited by a traced curve is enhanced compared to that elicited by distractor curves.[24] If the monkey makes an error and selects the wrong curve, it is the erroneously selected curve that elicits extra activity, implying that selection signals in V1 are related to the monkey's perceptual decision.[26–28] Previous studies demonstrated that V1 activity can also give insight into how information at multiple branch points (e.g., the central cue and the discriminant segments) is combined to reach a correct behavioral decision.[29,30] In the present paradigm, we therefore examined how the central cue and the discriminant segments jointly determine V1 activity. We configured the stimulus such that the V1 RF fell on the skeleton (Figure 7A). Specifically, the element in the RF now was the distal contour element connecting the discriminant segment to one of the purple circles. This distal contour element in the V1 RF was visible throughout the trial, and it was identical across all stimuli (Figure 7A). Hence, any modulation of V1 activity in this experiment arises due to contextual influences originating outside the RF, including the effects of attentional selection.

We recorded V1 activity at 25 recording sites in monkey M (22,016 trials in 9 sessions) and at 13 sites in monkey D (14,604 trials in 16 sessions; a hardware problem caused loss of neuronal data so that 7,004 trials in 8 sessions remained). Figures 7C–7E illustrate the neuronal data for monkey M (results in monkey D were similar, but we did not pool across monkeys because of the distinct event timing; see Figure S3).

In the pre-cue condition, the appearance of the skeleton's contour element in the RF triggered a visually driven response (Figures 7A–7C). The cue was presented at the same time so that the monkey knew which of the curve pairs was task relevant. Upon the appearance of the discriminant segments (t = 0 and blue horizontal line in Figure 7C; see also Figure S3A), the activity elicited by the distal contour element increased if it was connected to a discriminant segment (continuous green and black lines in Figures 7A–7C) compared to when it was not (dashed in Figures 7A–7C). We call this effect connectedness modulation (ConnMod) (green and gray filled regions in Figure 7C) and note that it reproduces the increased activity elicited by relevant curves in V1 during curve tracing[24] and other tasks that require contour integration.[31] ConnMod was determined in a time window from 100 to 300 ms after the appearance of the discriminant segments, and it was significant for both the cued (green area in Figures 7C and S3A; paired t test; monkey M, $p = 6.7 \cdot 10^{-23}$; monkey D, $p = 5.1 \cdot 10^{-9}$) and non-cued curve pairs (gray area in Figure 7C; monkey M, $p = 1.6 \cdot 10^{-17}$; monkey D, $p = 1.1 \cdot 10^{-5}$). Average ConnMod for the cued curve pair was 0.17 and 0.65 (normalized units; see STAR Methods) for monkeys M and D, respectively. ConnMod for the non-cued curve pair was weaker, on average of 0.05 and 0.27 for monkey M and D, respectively (paired t test; monkey M, $t_{24} = 25.6$, $p = 6.2 \cdot 10^{-19}$; monkey D, $t_{12} = 7.0$, $p = 1.3 \cdot 10^{-5}$). Apparently, the central cue amplified connectedness modulation for the relevant pair of curves and/or decreased it for the irrelevant pair. We measured the amplitude and time course of this attentional effect induced by the central cue by subtracting ConnMod for the irrelevant (cued) pair from that for the relevant (non-cued) pair (ΔConnMod; purple region in Figure 7C). This attentional effect on connectedness modulation appeared about 150 ms after the onset of the discriminant segments and lasted approximately 250 ms.

In the other trials, the cue was presented after the discriminant segments so that the monkey had to memorize all three discriminant segments. In these conditions, ConnMod was prominent during the phase in which the discriminant segments were visible ($p < 10^{-17}$ and $p < 10^{-5}$ in monkeys M and D, respectively; Figures 7C–7E). We next investigated the influence of cue timing. In the post-cue conditions, cueing of the curve pair within the RF increased ConnMod, after the discriminant segments had disappeared, especially if the COL was 0 ms (purple area in Figure 7D).

We calculated the average ΔConnMod in a 200-ms time window starting 100 ms after cue onset. In monkey M, the level of ΔConnMod decreased for longer COLs, from 0.063 at a COL of 0 ms to 0.026 at a COL of 176 ms (paired t test; $p = 2.6 \cdot 10^{-8}$; $t_{24} = 8.1$). Hence, the influence of the cue on connectedness modulation declines when the iconic memory trace subsides. We obtained similar results in monkey D with a ΔConnMod of 0.167 at a COL of 0 ms and a decreased value of −0.050 at a COL of 117 ms (the longest COL tested in this monkey; $p = 7.3 \cdot 10^{-4}$; $t_{12} = 4.5$).

To assess how well the influence of cue timing on neuronal responses (ΔConnMod) matched the influence on behavioral performance (d-prime), we performed a linear regression (Figure 7F). The match was very good (R^2 of 80.6% and $p = 0.039$ in monkey M; $R^2 = 93.1\%$ and $p = 0.008$ in monkey D). These results, taken

CellPress
OPEN ACCESS

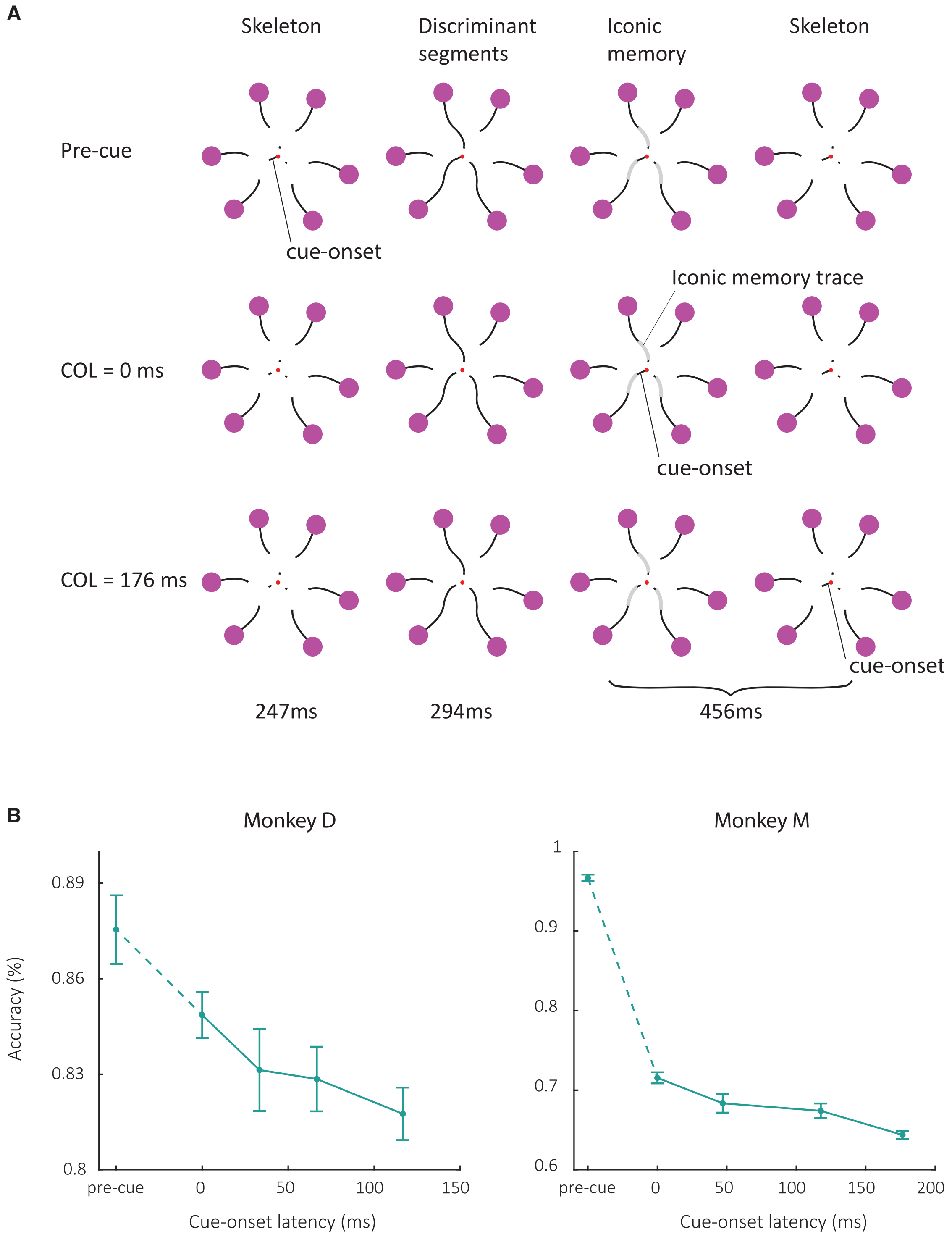

Figure 6. Varying the timing of the central cue

(A) In the pre-cue condition, the central cue was presented 50 ms before the onset of the discriminant segments and remained visible throughout the trial. Gray segments represent the iconic memory trace in the brain (not presented on the screen). In the COL = 0 condition, the central cue is presented while the iconic memory trace is still available. In the COL = 176 condition, the central cue is presented after iconic memory has decayed.

(B) Accuracy of monkey D (left) and M (right) as function of COL. Error bars represent SEM across sessions.

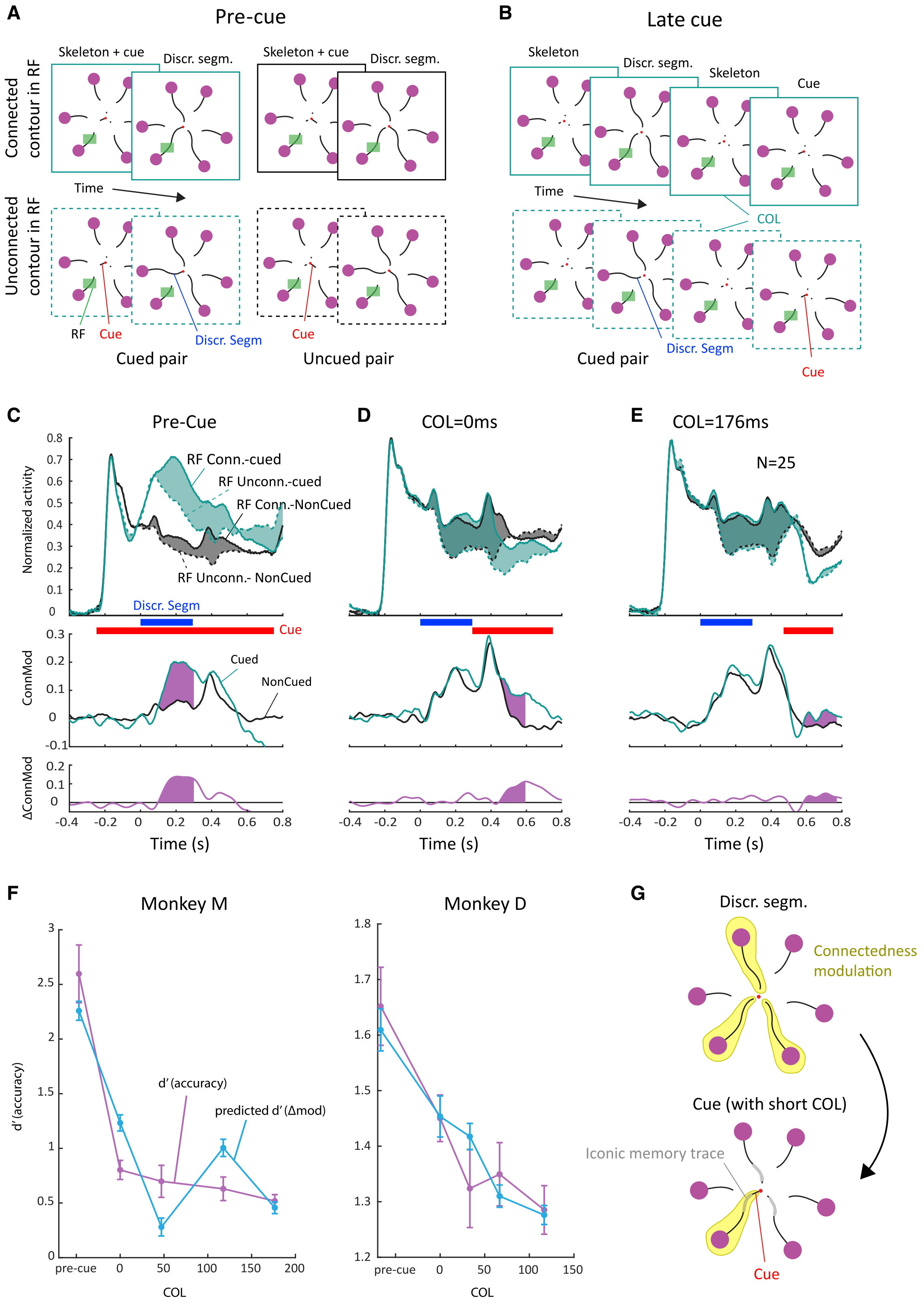

(legend on next page)

together, indicate that cues presented during the iconic memory phase (at a COL of 0 ms) improve the monkeys' accuracy and cause an increase in attentional selection signals in V1. Apparently, the cue was combined with the iconic memory trace of the discriminant segment to specifically increase the activity of V1 neurons with a RF close to the relevant purple circle (Figure 7G). However, once the iconic memory has decayed, the cue had little influence on the connectedness modulation and hardly benefitted the monkeys' performance.

DISCUSSION

Previous research demonstrated that iconic memory resembles a fading sensory trace of a visual image[7,22] with a rich content that can be used as if the stimulus were still visible.[32] We reproduced critical properties of the classic Sperling paradigm in monkeys, using an adapted curve-tracing paradigm (Figure 1A). We demonstrated that properties of iconic memory correspond to the decaying V1 activity that occurs after a stimulus has disappeared. The monkeys had to memorize a number of discriminant segments in a whole report version of the paradigm, in which we presented a central cue at a late time point. The monkeys' accuracy increased for longer stimulus durations, but it did not reach 100%, in accordance with a limited memory capacity of less than 2 items of the two monkeys.

We measured the worth, an estimate of the additional viewing time to which iconic memory is equivalent, by comparing the monkey's accuracy in trials with and without a mask.[22] The mask caused a rightward shift of the psychometric function, indicating that iconic memory was worth 61 ms in monkey D (95% confidence interval [39 ms, 91 ms]) and 66 ms in monkey M (95% confidence interval [47 ms, 86 ms]), which is slightly less than the worth reported in humans (70–100 ms).[22] The late, decaying activity after stimulus offset[21] in V1 accounted for the properties of iconic memory. The V1 information about the location of the discriminant segment was overridden by the metacontrast mask, which invariably evoked a strong, non-additive neuronal response.[16,19] In non-masked trials, the extra V1 activity did not strongly depend on stimulus duration and was therefore equivalent to a fixed amount extra viewing time. Accordingly, we could estimate a neuronal worth in V1 as the extra viewing time necessary to compensate for masking and obtained values of 44 ms and 70 ms in the two monkeys. These values were compatible with the behaviorally determined worths, supporting the view that the decaying V1 activity after stimulus offset provides a neuronal correlate of iconic memory. This view was supported by an analysis of individual trials. A higher level of decaying V1 activity after stimulus offset predicted a higher accuracy but only if monkeys reported the discriminant segment that had fallen in the RF of the V1 neurons.

The hypothesis that the decaying V1 response corresponds to iconic memory was further supported when we assessed the decay of iconic memory by varying the delay between stimulus offset and the mask. In accordance with human psychophysics,[22,33] we observed that the accuracy of the monkeys initially improved quickly when the MOL was prolonged but that further increases gave rise to diminishing returns. The shape of the behavioral decay function was correlated to the extra V1 activity, signaling the location of the discriminant segment when the mask was delayed (Figure 5D). The results of the first two paradigms, taken together, revealed a remarkable similarity between the properties of iconic memory and the decay of neuronal information in V1. In accordance with the literature on iconic memory, the worth corresponded to a constant amount of extra viewing time, reflected by horizontal shifts of the psychometric and neurometric functions (Figures 2, 3C, and 3D). The decay experiment extended these findings by showing that the decay of V1 activity also correlates with the partial worth when the mask is postponed. Together, these results indicate that the gradual decay of V1 activity after the disappearance of a visual stimulus is a plausible neural correlate of iconic memory. We are not aware of alternative neuronal mechanisms for iconic memory, but it would be useful if future studies could examine the influence of causal manipulations, like, for example, optogenetic silencing, on the behavioral worth. We note, however, that complete optogenetic silencing of cortical activity in monkeys is still hard to achieve with current technology.

A recent psychophysical study suggested that items in iconic memory do not decay gradually but abruptly, object-by-object, based on an analysis of how precise the features of a briefly presented stimulus can be reproduced later.[34] According to this view, the gradual decay of activity observed here could be caused by averaging across trials, whereas an abrupt decay at different time points should be apparent when analyzing

Figure 7. Interactions between iconic memory and central cue timing

(A) In the pre-cue condition, the central cue appeared before the discriminant segment. The neurons' RF fell on a distal contour of the skeleton (green rectangle), which was either connected to the discriminant segment (frames with continuous lines) or not (dashed). The pair of curves with the RF could be cued (green) or non-cued (black) with the central line element.

(B) In the post-cueing conditions, the central cue appeared after the discriminant segments had disappeared. We here only illustrate conditions in which the curve pair with the RFs was cued.

(C–E) Average V1 activity across 25 recording sites in monkey M in the pre-cue condition (C), COL = 0 condition (D), and COL = 176 ms (E). Time zero corresponds to the onset of the three discriminant segments. Note that the contour element in the RF appeared at −250 ms, simultaneously with the other parts of the stimulus skeleton and before the discriminant segments (see A). The contour element in the RF was either connected to the discriminant segment (continuous traces) or not (dashed traces). The pair of curves covering the RF could be cued centrally (green) or not (black). Responses were stronger if the contour in the RF was connected to the discriminant segment than if it was not. The extra activity is called ConnMod (green and gray areas) and represents a contextual influence from outside the RF. The middle panels show the time course of ConnMod for cued and non-cued curve pairs and lower panels the difference, ΔConnMod (purple area), which represents the cueing effect. The lower panels show the time course ΔConnMod (y axis), i.e., $\text{ConnMod}_{\text{cued}} - \text{ConnMod}_{\text{non-cued}}$.

(F) Behavioral d-prime based on the monkey's accuracy (magenta line, SEM across recording sessions). We used the average ΔConnMod to estimate d-prime using linear regression (blue curve). Error bars represent SEM across recording sites.

(G) Schematic representation of ConnMod when the central cue appears at a COL of 0 ms so that the iconic memory trace for the discriminant segments (gray) is still present and can boost connectedness modulation.

See also Figure S3.

individual trials. To provide some insight into this matter, we took advantage of the simultaneous recordings, allowing us to average across V1 sites instead of across trials (Figure S4). The results indicate that V1 activity decays gradually and with a similar time course across single trials. They do not support the abrupt decay of iconic memories at different time points, although more work may be needed to fully resolve the debate.

Lastly, we investigated how cues interact with iconic memory to increase the monkeys' accuracy. We created a partial report version of the paradigm by presenting the central cue at different delays after the discriminant segments. Just as in Sperling's paradigm,[3] the accuracy improved if the delay between the discriminant segments and the cue was short, as if the cue was just in time to capture the iconic memory trace.[35] In the partial report task, we measured a strong modulatory influence on V1 activity elicited by contour elements between the discriminant segment and the eye-movement targets. These contour elements were part of the skeleton and therefore visible during the entire trial. In accordance with previous work,[29,36] these distal contours elicited "connectedness modulation" in V1; more activity if they were connected to the discriminant segment than if they were not (Figure 7G, top). Interestingly, the central cue interacted with the connectedness modulation. Early cues presented before the discriminant segments resulted in stronger modulation for the cued curve pair than for non-cued pairs. Cues that were presented later than the discriminant segments also increased the connectedness modulation but only during the iconic memory phase. The influence of the cue on connectedness modulation accounted for the monkey's accuracy in the task, because early cues caused extra connectedness modulation for the relevant pair of curves, as if the discriminant segments were still visible (Figure 7G, bottom), whereas late cues did not.

The difference in accuracy between early and late cueing trials of the partial report task was 3% and 8% in the two monkeys, which may seem modest if compared to the results of Sperling.[3] This difference between results is presumably caused by several factors. First, the monkeys reported the position of one of two discriminant segments of the cued pair of curves, with a chance level of 50%. In contrast, the subjects of Sperling's task could report up to four letters or digits, and the chance level of reporting these items was therefore much lower. Hence, the dynamic range of Sperling's outcome measure was much larger. Second, we tested relatively short delays between the offset of the discriminant segments and the onset of the cue, given the monkeys' impatience when we tried longer delays. It is conceivable that their accuracy would have decreased further had we used longer delays. Importantly, the influence of cue timing observed by us is comparable to that in a previous study on iconic memory in humans[7] that also used cueing and observed a decrease in accuracy of 7% when the cue was postponed by 150 ms.

Although we here focused on V1, the present results do not exclude the involvement of other cortical and subcortical brain regions in iconic memory. The iconic decay function of V1 activity is longer than the time constant of single cortical neurons, which is only a few milliseconds.[37] The decay function therefore must reflect properties of recurrent loops, which exist within V1[38] and also between V1 and other brain structures. Of interest in this context is a study in mice demonstrating that lateral geiculate nucleus (LGN) silencing abolishes V1 activity within approximately 10 ms.[39] This result suggests that V1 in interaction with higher cortical areas does, by itself, not sustain activity long enough to account for the shape of iconic decay function but that it depends on loops that include the LGN and possibly other subcortical structures.

However, our results do also not exclude contributions of extrastriate visual cortical areas V2, V3, and V4, which have their own recurrent loops with the thalamus and exhibit a decaying response upon the disappearance of a visual stimulus, resembling the V1 response.[17,40,41] The properties of offset responses in extrastriate cortex remain to be studied systematically, however (with the exception of a study[42] in which the RF stimulus was followed by another stimulus, causing a masking effect).

Neuronal responses in even higher areas, such as the inferotemporal cortex, last hundreds of milliseconds[43,44] and sometimes even up to seconds[45,46] after stimulus offset. This is longer than the decay time of iconic memory, making it less likely that neurons in these areas account for the properties of iconic memory. When a stimulus is followed by a mask, however, the activity of inferotemporal neurons is quickly overwritten.[18,43,44] Neurons in the inferotemporal cortex presumably integrate visual information from lower areas, which also explains why their activity depends less on the precise duration of a brief visual stimulus.[18] They may contribute to a form of memory called "fragile short-term visual memory,"[12,47,48] which encodes less detail and lasts seconds but can, just like iconic memory, be interrupted by a mask.

The most stable form of short-term visual memory is working memory. Working memory is resistant to masking,[49,50] but it has a limited capacity of only a few items.[51,52] Neurons in the frontal[53] and parietal cortex[54] as well as in the medial temporal lobe[45,55] play a prominent role in the maintenance of working memory representations, encoding previously presented stimuli with persistent activity. Although these working memory signals are weaker in low-level visual areas,[56,57] their involvement in the maintenance of working memories may be task dependent.[1] It is remarkable that recent studies demonstrated that the maintenance of the persistent activity in the frontal cortex also depends on loops between cortex and the thalamus,[58] just like we mentioned regarding iconic memory in V1. It is therefore tempting to speculate that the time constants of these thalamocortical loops increase from low-level to higher level areas.

These considerations, taken together, support the idea that processing timescales increase when ascending the visual cortical hierarchy.[59,60] Higher areas contribute to increasingly stable forms of visual short-term memory, going from iconic memory in low-level areas to fragile visual short-term memory in mid-level visual areas and working memory in association areas.[32] The accompanying decrease in storage capacity implies that not all iconic memories can be stored as working memories. As a result, cues that are presented when iconic memory is still active can promote the storage of relevant information in a durable, reportable form in the higher areas, whereas cues presented later cannot, because iconic memory has decayed.

In summary, our results demonstrate how the decaying response of neurons in low-level visual areas can account for the worth and decay of iconic memory. Future research can take advantage of these findings to further explore how iconic memories are transformed into more stable forms of memory,

such as working memory. Indeed, the considerations above inspire an important question for future work: do these three forms of memory, iconic memory, fragile memory, and working memory, provide a comprehensive account, or are they the first approximation to a rich hierarchy of gradually increasing time constants in higher cortical areas?

STAR★METHODS

Detailed methods are provided in the online version of this paper and include the following:

- KEY RESOURCES TABLE
- RESOURCE AVAILABILITY
  - Lead contact
  - Materials availability
  - Data and code availability
- EXPERIMENTAL MODEL AND SUBJECT DETAILS
- METHOD DETAILS
  - Surgical procedures and training
  - Electrophysiology
  - Behavioral task and stimuli
- QUANTIFICATION AND STATISTICAL ANALYSIS
  - Estimation of behavioral and neuronal worth
  - Behavioral d-prime
  - Relation between the magnitude of the decaying response and accuracy
  - Likelihood ratio test for performance on decay paradigm
  - Connectedness modulation

SUPPLEMENTAL INFORMATION

Supplemental information can be found online at https://doi.org/10.1016/j.cub.2021.09.052.

ACKNOWLEDGMENTS

We thank Kor Brandsma and Anneke Ditewich for biotechnical support. The work was supported by the European Union's Horizon 2020 and FP7 Research and Innovation Program (grant agreements 720270 and 785907 "Human Brain Project SGA1 and SGA2"; ERC grant agreement 339490 "Cortic_al_gorithms") and INTENSE (NWO Crossover, no. 17619).

AUTHOR CONTRIBUTIONS

C.W., U.H.S., R.R.M.T., and P.R.R. developed the task. R.R.M.T. and U.H.S. recorded the electrophysiological data. R.R.M.T., M.W.S., and C.W. analyzed the data. R.R.M.T., U.H.S., and P.R.R. wrote the paper.

DECLARATION OF INTERESTS

P.R.R. is founder and shareholder of Phosphoenix, a company that aims to develop a visual brain prosthesis for blind people.

Received: November 26, 2020
Revised: July 13, 2021
Accepted: September 21, 2021
Published: October 14, 2021

REFERENCES

1. Christophel, T.B., Klink, P.C., Spitzer, B., Roelfsema, P.R., and Haynes, J.D. (2017). The distributed nature of working memory. Trends Cogn. Sci. *21*, 111–124.
2. Curtis, C.E., and D'Esposito, M. (2003). Persistent activity in the prefrontal cortex during working memory. Trends Cogn. Sci. *7*, 415–423.
3. Sperling, G. (1960). The information available in brief visual presentations. Psychol. Monogr. Gen. Appl. *74*, 1–29.
4. Averbach, E., and Coriell, A.S. (1961). Short-term memory in vision. Bell Syst. Tech. J. *40*, 309–328.
5. Phillips, W.A. (1974). On the distinction between sensory storage and short-term visual memory. Percept. Psychophys. *16*, 283–290.
6. Coltheart, M. (1980). Iconic memory and visible persistence. Percept. Psychophys. *27*, 183–228.
7. Graziano, M., and Sigman, M. (2008). The dynamics of sensory buffers: geometric, spatial, and experience-dependent shaping of iconic memory. J. Vis. *8*, 9.1–13.
8. Luck, S.J., and Vogel, E.K. (1997). The capacity of visual working memory for features and conjunctions. Nature *390*, 279–281.
9. Luck, S.J., and Vogel, E.K. (2013). Visual working memory capacity: from psychophysics and neurobiology to individual differences. Trends Cogn. Sci. *17*, 391–400.
10. Ma, W.J., Husain, M., and Bays, P.M. (2014). Changing concepts of working memory. Nat. Neurosci. *17*, 347–356.
11. Fallon, S.J., Zokaei, N., and Husain, M. (2016). Causes and consequences of limitations in visual working memory. Ann. N Y Acad. Sci. *1369*, 40–54.
12. Sligte, I.G., Scholte, H.S., and Lamme, V.A.F. (2009). V4 activity predicts the strength of visual short-term memory representations. J. Neurosci. *29*, 7432–7438.
13. Long, G.M. (1980). Iconic memory: a review and critique of the study of short-term visual storage. Psychol. Bull. *88*, 785–820.
14. Duysens, J., Orban, G.A., Cremieux, J., and Maes, H. (1985). Visual cortical correlates of visible persistence. Vision Res. *25*, 171–178.
15. Keysers, C., Xiao, D.K., Földiák, P., and Perrett, D.I. (2001). The speed of sight. J. Cogn. Neurosci. *13*, 90–101.
16. Lamme, V.A.F., Zipser, K., and Spekreijse, H. (2002). Masking interrupts figure-ground signals in V1. J. Cogn. Neurosci. *14*, 1044–1053.
17. van Vugt, B., Dagnino, B., Vartak, D., Safaai, H., Panzeri, S., Dehaene, S., and Roelfsema, P.R. (2018). The threshold for conscious report: signal loss and response bias in visual and frontal cortex. Science *360*, 537–542.
18. Keysers, C., Xiao, D.K., Földiák, P., and Perrett, D.I. (2005). Out of sight but not out of mind: the neurophysiology of iconic memory in the superior temporal sulcus. Cogn. Neuropsychol. *22*, 316–332.
19. Macknik, S.L., and Livingstone, M.S. (1998). Neuronal correlates of visibility and invisibility in the primate visual system. Nat. Neurosci. *1*, 144–149.
20. Rolls, E.T., Tovée, M.J., and Panzeri, S. (1999). The neurophysiology of backward visual masking: information analysis. J. Cogn. Neurosci. *11*, 300–311.
21. Zylberberg, A., Dehaene, S., Mindlin, G.B., and Sigman, M. (2009). Neurophysiological bases of exponential sensory decay and top-down memory retrieval: a model. Front. Comput. Neurosci. *3*, 4.
22. Loftus, G.R., Duncan, J., and Gehrig, P. (1992). On the time course of perceptual information that results from a brief visual presentation. J. Exp. Psychol. Hum. Percept. Perform. *18*, 530–549, discussion 550–561.
23. Lu, Z.-L., Neuse, J., Madigan, S., and Dosher, B.A. (2005). Fast decay of iconic memory in observers with mild cognitive impairments. Proc. Natl. Acad. Sci. USA *102*, 1797–1802.
24. Roelfsema, P.R., Lamme, V.A.F., and Spekreijse, H. (1998). Object-based attention in the primary visual cortex of the macaque monkey. Nature *395*, 376–381.

25. Scholte, H.S., Spekreijse, H., and Roelfsema, P.R. (2001). The spatial profile of visual attention in mental curve tracing. Vision Res. *41*, 2569–2580.

26. Roelfsema, P.R., and Spekreijse, H. (2001). The representation of erroneously perceived stimuli in the primary visual cortex. Neuron *31*, 853–863.

27. Pooresmaeili, A., Poort, J., and Roelfsema, P.R. (2014). Simultaneous selection by object-based attention in visual and frontal cortex. Proc. Natl. Acad. Sci. USA *111*, 6467–6472.

28. Nienborg, H., and Roelfsema, P.R. (2015). Belief states as a framework to explain extra-retinal influences in visual cortex. Curr. Opin. Neurobiol. *32*, 45–52.

29. Lorteije, J.A.M., Zylberberg, A., Ouellette, B.G., De Zeeuw, C.I., Sigman, M., and Roelfsema, P.R. (2015). The formation of hierarchical decisions in the visual cortex. Neuron *87*, 1344–1356.

30. Zylberberg, A., Lorteije, J.A.M., Ouellette, B.G., De Zeeuw, C.I., Sigman, M., and Roelfsema, P. (2017). Serial, parallel and hierarchical decision making in primates. eLife *6*, 1–9.

31. Li, W., Piëch, V., and Gilbert, C.D. (2006). Contour saliency in primary visual cortex. Neuron *50*, 951–962.

32. Rensink, R.A. (2014). Limits to the usability of iconic memory. Front. Psychol. *5*, 971.

33. Gegenfurtner, K.R., and Sperling, G. (1993). Information transfer in iconic memory experiments. J. Exp. Psychol. Hum. Percept. Perform. *19*, 845–866.

34. Pratte, M.S. (2018). Iconic memories die a sudden death. Psychol. Sci. *29*, 877–887.

35. Turvey, M.T., and Kravetz, S. (1970). Retrieval from iconic memory with shape as the selection criterion. Percept. Psychophys. *8*, 171–172.

36. Houtkamp, R., Spekreijse, H., and Roelfsema, P.R. (2003). A gradual spread of attention during mental curve tracing. Percept. Psychophys. *65*, 1136–1144.

37. Kuhn, A., Aertsen, A., and Rotter, S. (2004). Neuronal integration of synaptic input in the fluctuation-driven regime. J. Neurosci. *24*, 2345–2356.

38. Douglas, R.J., Koch, C., Mahowald, M., Martin, K.A., and Suarez, H.H. (1995). Recurrent excitation in neocortical circuits. Science *269*, 981–985.

39. Reinhold, K., Lien, A.D., and Scanziani, M. (2015). Distinct recurrent versus afferent dynamics in cortical visual processing. Nat. Neurosci. *18*, 1789–1797.

40. Luck, S.J., Chelazzi, L., Hillyard, S.A., and Desimone, R. (1997). Neural mechanisms of spatial selective attention in areas V1, V2, and V4 of macaque visual cortex. J. Neurophysiol. *77*, 24–42.

41. Reynolds, J.H., Chelazzi, L., and Desimone, R. (1999). Competitive mechanisms subserve attention in macaque areas V2 and V4. J. Neurosci. *19*, 1736–1753.

42. Bair, W., Cavanaugh, J.R., Smith, M.A., and Movshon, J.A. (2002). The timing of response onset and offset in macaque visual neurons. J. Neurosci. *22*, 3189–3205.

43. Kovács, G., Vogels, R., and Orban, G.A. (1995). Cortical correlate of pattern backward masking. Proc. Natl. Acad. Sci. USA *92*, 5587–5591.

44. Rolls, E.T., and Tovee, M.J. (1994). Processing speed in the cerebral cortex and the neurophysiology of visual masking. Proc. Biol. Sci. *257*, 9–15.

45. Chelazzi, L., Miller, E.K., Duncan, J., and Desimone, R. (1993). A neural basis for visual search in inferior temporal cortex. Nature *363*, 345–347.

46. Yakovlev, V., Fusi, S., Berman, E., and Zohary, E. (1998). Inter-trial neuronal activity in inferior temporal cortex: a putative vehicle to generate long-term visual associations. Nat. Neurosci. *1*, 310–317.

47. Sligte, I.G., Scholte, H.S., and Lamme, V.A.F. (2008). Are there multiple visual short-term memory stores? PLoS ONE *3*, e1699.

48. Sligte, I.G., Vandenbroucke, A.R.E., Scholte, H.S., and Lamme, V.A.F. (2010). Detailed sensory memory, sloppy working memory. Front. Psychol. *1*, 175.

49. Miller, E.K., Erickson, C.A., and Desimone, R. (1996). Neural mechanisms of visual working memory in prefrontal cortex of the macaque. J. Neurosci. *16*, 5154–5167.

50. Warden, M.R., and Miller, E.K. (2010). Task-dependent changes in short-term memory in the prefrontal cortex. J. Neurosci. *30*, 15801–15810.

51. Cowan, N. (2001). The magical number 4 in short-term memory: a reconsideration of mental storage capacity. Behav. Brain Sci. *24*, 87–114, discussion 114–185.

52. Oberauer, K. (2002). Access to information in working memory: exploring the focus of attention. J. Exp. Psychol. Learn. Mem. Cogn. *28*, 411–421.

53. Rainer, G., Asaad, W.F., and Miller, E.K. (1998). Selective representation of relevant information by neurons in the primate prefrontal cortex. Nature *393*, 577–579.

54. Gnadt, J.W., and Andersen, R.A. (1988). Memory related motor planning activity in posterior parietal cortex of macaque. Exp. Brain Res. *70*, 216–220.

55. Gelbard-Sagiv, H., Mukamel, R., Harel, M., Malach, R., and Fried, I. (2008). Internally generated reactivation of single neurons in human hippocampus during free recall. Science *322*, 96–101.

56. Mendoza-Halliday, D., Torres, S., and Martinez-Trujillo, J.C. (2014). Sharp emergence of feature-selective sustained activity along the dorsal visual pathway. Nat. Neurosci. *17*, 1255–1262.

57. van Kerkoerle, T., Self, M.W., and Roelfsema, P.R. (2017). Layer-specificity in the effects of attention and working memory on activity in primary visual cortex. Nat. Commun. *8*, 13804.

58. Guo, Z.V., Inagaki, H.K., Daie, K., Druckmann, S., Gerfen, C.R., and Svoboda, K. (2017). Maintenance of persistent activity in a frontal thalamocortical loop. Nature *545*, 181–186.

59. Chaudhuri, R., Knoblauch, K., Gariel, M.A., Kennedy, H., and Wang, X.J. (2015). A large-scale circuit mechanism for hierarchical dynamical processing in the primate cortex. Neuron *88*, 419–431.

60. Murray, J.D., Bernacchia, A., Freedman, D.J., Romo, R., Wallis, J.D., Cai, X., Padoa-Schioppa, C., Pasternak, T., Seo, H., Lee, D., and Wang, X.J. (2014). A hierarchy of intrinsic timescales across primate cortex. Nat. Neurosci. *17*, 1661–1663.

61. Self, M.W., Kooijmans, R.N., Supèr, H., Lamme, V.A., and Roelfsema, P.R. (2012). Different glutamate receptors convey feedforward and recurrent processing in macaque V1. Proc. Natl. Acad. Sci. USA *109*, 11031–11036.

62. Chen, X., Possel, J.K., Wacongne, C., van Ham, A.F., Klink, P.C., and Roelfsema, P.R. (2017). 3D printing and modelling of customized implants and surgical guides for non-human primates. J. Neurosci. Methods *286*, 38–55.

63. Supèr, H., and Roelfsema, P.R. (2005). Chronic multiunit recordings in behaving animals: advantages and limitations. Prog. Brain Res. *147*, 263–282.

64. Cohen, M.R., and Maunsell, J.H.R. (2009). Attention improves performance primarily by reducing interneuronal correlations. Nat. Neurosci. *12*, 1594–1600.

65. Palmer, C., Cheng, S.Y., and Seidemann, E. (2007). Linking neuronal and behavioral performance in a reaction-time visual detection task. J. Neurosci. *27*, 8122–8137.

66. Ramachandran, V.S., and Cobb, S. (1995). Visual attention modulates metacontrast masking. Nature *373*, 66–68.

67. DeCarlo, L.T. (2012). On a signal detection approach to *m*-alternative forced choice with bias, with maximum likelihood and Bayesian approaches to estimation. J. Math. Psychol. *56*, 196–207.

68. Prins, N., and Kingdom, F.A.A. (2018). Applying the model-comparison approach to test specific research hypotheses in psychophysical research using the Palamedes Toolbox. Front. Psychol. *9*, 1250.

CellPress
OPEN ACCESS

STAR★METHODS

KEY RESOURCES TABLE

REAGENT or RESOURCE	SOURCE	IDENTIFIER
Experimental Models: Organisms/Strains		
Macaca Mulatta	Biological Primate Research Center	https://bprc.nl/nl/home
Software and algorithms		
MATLAB R2017b	Mathworks Inc.	https://www.mathworks.com/products/matlab.html
Electrophysiological data	OSF	https://doi.org/10.17605/OSF.IO/K9JCF
Behavioral data	OSF	https://doi.org/10.17605/OSF.IO/K9JCF
Tucker-Davis Technologies recording system	Tucker-Davis Technologies	RRID:SCR_006495
Infrared video eye tracker	Thomas Recording GmbH	ET49 https://www.thomasrecording.com/et-49-230hz

RESOURCE AVAILABILITY

Lead contact

Further information and requests for resources and reagents should be directed to and will be fulfilled by the Lead Contact, Pieter Roelfsema (p.roelfsema@nin.knaw.nl).

Materials availability

This study did not generate new unique reagents.

Data and code availability

The datasets generated during this study are publicly available at Open Science Framework: https://osf.io/k9jcf/ (https://doi.org/10.17605/OSF.IO/K9JCF). Correspondence and requests for materials can be sent to PRR (p.roelfsema@nin.knaw.nl).

EXPERIMENTAL MODEL AND SUBJECT DETAILS

All animal procedures complied with the NIH Guide for Care and Use of Laboratory Animals, and were approved by the institutional animal care and use committee of the Royal Netherlands Academy of Arts and Sciences. Two male macaque monkeys (aged 9 and 12 at the start of the experiments) participated in the experiment. The monkeys were socially housed in pairs in a specialized primate facility with natural daylight, controlled humidity and temperature. The home-cage was a large floor-to-ceiling cage that allowed natural climbing and swinging behavior. The cage had a solid floor, covered with sawdust and was enriched with toys and foraging items. The diet consisted of monkey chow supplemented with fresh fruit. The access to fluid was controlled, according to a carefully designed regime for fluid uptake. During weekdays the animals received diluted fruit juice in the experimental set-up upon correctly performed trials. We ensured that the animals drank sufficient fluid in the set-up and supplemented extra fluid after the recording session if the monkeys did not drink enough. In the weekend the animals received at least 700ml of water in the home-cage supplied in a drinking bottle. The animals were regularly checked by veterinary staff and animal caretakers and their weight and general appearance were recorded in an electronic logbook on a daily basis during fluid-control periods.

METHOD DETAILS

Surgical procedures and training

We implanted both monkeys with a titanium head-post (Crist instruments) under aseptic conditions and general anesthesia as reported previously[61,62]. The monkeys were first trained to fixate on a 0.5 diameter fixation dot and hold their eyes within a small fixation window (1.1 diameter). They then underwent a second operation to implant arrays of 5x5 micro-electrodes (Blackrock Microsystems) in opercular V1. The inter-electrode spacing of the arrays was 400 μm. The animals were later trained to perform the iconic memory task. We first obtained the data for the worth paradigm, after which the monkeys were trained on the slightly altered tasks for the decay and cueing experiments. We recorded neuronal activity from one array in monkey D and two arrays in monkey M.

Electrophysiology

We recorded MUA using a PZ2 preamplifier and a RZ2-8 signal processor (Tucker Davis Technology). The signal was referenced to a subdural electrode and digitized at 24.4 kHz. It was band-pass filtered (2nd order Butterworth filter, 500Hz-5KHz) to isolate high-frequency (spiking) activity. This signal was rectified (negative becomes positive) and low-pass filtered (corner frequency = 200Hz) to produce multi-unit activity (MUA), which is the envelope of the high-frequency activity[63]. MUA reflects the spiking of neurons within 100-150mm of the electrode and MUA population responses are very similar to those obtained by pooling across single units[61,63–65]. We used a video-camera based eye-tracker (Thomas Recording) to measure the eye position at a sampling frequency of 1017Hz in monkey D and 508Hz in monkey M. We mapped the RFs of neurons at all recording sites using a moving light bar stimulus, as described previously[63].

We removed artifacts, i.e., trials with extreme MUA, using an iterative z-scoring procedure (values higher than 3 were removed). If z-scores higher than 15 remained, the process was repeated, leading to the removal of less than 0.4% of all the trials. If necessary, we used a second order Butterworth notch to filter out the refresh frequency of the monitor (55-65Hz). To normalize MUA, we subtracted the spontaneous activity level in a time window from 250 to 50ms prior to the onset of the skeleton and dividing by the peak response after LOWESS smoothing (26ms window). We only included recording sites with a signal-to-noise higher than 1. The signal-to-noise ratio was computed by dividing the peak of the smoothed response by the standard deviation of the spontaneous activity level across trials.

Behavioral task and stimuli

Stimuli were presented on CRT monitors (Dell) at a refresh rate of 85Hz for monkey D and 60Hz for monkey M, with a resolution of 1024x768 pixels, viewed from a distance of 44cm (monkey D) or 56cm (monkey M). The monitor for monkey D was 40 × 30cm yielding a field-of-view of 48.9° x 37.6°, and the monitor for monkey M was 38.5 × 29 cm yielding a field-of-view of 37.9° x 29°. All stimuli were created using the COGENT graphics toolbox (developed by John Romaya at the LON at the Well-come Department of Imaging Neuroscience) running in MATLAB (Mathworks Inc.). Monkeys performed a modified version of a curve-tracing task[24] (Figures 1B–1D). A trial started with a 300ms period of fixation, after which we presented the skeleton of the stimulus. The skeleton consisted of 3 pairs of 2 saccade targets (purple circles with a diameter of 2.5° at 6° eccentricity for monkey D and a diameter of 1.5° at 4.7° eccentricity for monkey M) that were arranged around the fixation point. The contour elements were white (68 cd.m^{-2} for monkey D and 72 cd.m^{-2} for monkey M) on a gray background (25 cd.m^{-2}). There were three discriminant segments, which were briefly presented. The purple circle connected to the discriminant segment was the target for an eye movement. After a delay during which only the skeleton was visible, a central contour element appeared, cueing the monkey to make an eye movement to the purple circle of that pair that had been connected to the discriminant segment. Hence, the monkey had to remember all three discriminant segments. In the response period, 750ms after the appearance of the discriminant segments, the fixation point changed from red to green, cueing the monkey to make an eye movement (Figure 1). To avoid always presenting the exact same stimulus skeleton, we rotated the entire stimulus display across trials in 4 steps of 5 degrees, but ensured that the discriminant segment was in the RF in all trials. We pooled across these rotations in the analysis.

We used 3 slightly different versions of the paradigm to measure the worth, decay and the influence of cue timing. In the worth experiment, we blocked iconic memory in half of the trials by presenting a meta-contrast mask for 100ms immediately after the offset of the discriminant segments (Figure 1D)[66] and we varied the duration of these segments across trials (58, 105, 152, 200, or 247ms). In the decay experiment, we chose a duration of the discriminant segments that yielded a large accuracy difference between the mask and no mask conditions, based on the monkey's performance in the worth experiment. We fixed this duration (83ms for monkey D and 153ms for monkey M) and varied the MOL, which is the time between the offset of the discriminant segments and the onset of the mask (0, 17, 50, 83, or 183ms for monkey D and 0, 24, 59, 94, or 177ms for monkey M). The mask was presented on all trials. In the cue timing experiment, there was no mask and the stimulus duration was fixed (183ms for monkey D, 294ms for monkey M), while we varied the cue onset latency (COL), i.e., the time between the offset of the discriminant segments and the onset of the central cue (0, 33, 67, 117ms for monkey D and 0, 47, 117, or 176ms for monkey M). There also was a pre-cue condition in which the central cue was presented as soon as the skeleton appeared (247ms before the onset of the discriminant segments) and remained visible throughout the entire duration of the trial. In all experiments, the duration of the delay was set so that the time between the onset of the discriminant segments and the cue to make a saccade (change in the color of the fixation point) was 750ms.

We recorded behavioral and electrophysiological data simultaneously. In the worth experiment, we recorded 15,133 trials across 13 recording sessions in monkey D and 20,891 trials across 7 sessions in monkey M. In the partial worth experiment, we collected 10,778 trials across 10 sessions in monkey D and 21,522 trials across 9 sessions in monkey M. In both the worth and partial worth experiments we obtained useful data from 20 recording sites in monkey D and 18 recording sites in monkey M. In the partial report experiment we collected 7,004 trials across 8 sessions in monkey D and 22,016 trials across 9 sessions in monkey M. We recorded an additional 6 sessions of data in monkey D, but due to a hardware problem the neuronal data from these sessions were not usable. Hence, the behavioral data of the partial report experiment in monkey D contained 14,604 trials recorded during 14 sessions. We obtained data from 13 recording sites in monkey D and 25 recording sites in monkey M. The trial numbers were equally distributed across conditions. To estimate the reliability of the behavioral performance, we calculated the SEM of the accuracy across sessions, which always were on different days.

CellPress
OPEN ACCESS

QUANTIFICATION AND STATISTICAL ANALYSIS

Estimation of behavioral and neuronal worth

To measure the worth of iconic memory we fitted a sigmoidal curve with 3 free parameters to the monkeys' accuracy as a function of stimulus duration (Figure 2) using non-linear least-squares regression:

$$f(x) = 0.5 + \frac{(Max_{Acc} - 0.5)}{1 + e^{-\beta(\mu - x)}}$$

Where x is time in ms, Max_{Acc} determines the maximum accuracy reached for stimuli of very long duration, β the slope of the curve, and μ the stimulus duration at the inflection point. We constrained the curves for the masked and non-masked trials to have the same slope, assuming that iconic memory does not depend on stimulus duration[22]. They also had the same maximum because the accuracy is expected to reach a ceiling for long durations, in which case iconic memory has no effect. The worth is estimated as the horizontal shift between the two curves.

To compute the neuronal worth, we examined the average MUA responses per monkey. For each stimulus duration, we computed $Resp_{Diff}$, the difference in activity elicited by the discriminant segment inside and outside the RF (Figures 3A and 3B bottom rows). We integrated the positive part of $Resp_{Diff}$ (colored area in Figures 3A and 3B bottom rows) and fit a General Linear Model with a single slope term (Figures 3C and 3D) for the conditions with and without a mask. We estimated the neuronal worth as the horizontal shift between these lines. We also repeated this analysis per recording site (insets in Figures 3C and 3D).

We used a bootstrapping procedure to estimate the variability of behavioral and neuronal worth estimates. For each condition (e.g., stimulus duration, mask on/off) we randomly selected, with replacement, the same number of trials. We repeated this procedure 1,000 times and recalculated the behavioral and neuronal worth, resulting in a distribution of worth estimates and used it to determine 95%-confidence intervals.

Behavioral d-prime

In the partial worth paradigm, we integrated the positive part of $Resp_{Diff}$ to estimate the behavioral d-prime, using linear regression. Behavioral d-prime was calculated from the accuracy as follows[67]:

$$d' = \sqrt{2}\,\Phi^{-1}(a)$$

Where a is the accuracy, and Φ^{-1} the inverse of the standard normal cumulative distribution.

Relation between the magnitude of the decaying response and accuracy

We used a SVM to investigate whether variations in the level of decaying activity in V1 across trials predict the monkey's accuracy. We trained the SVM to discriminate between trials with the discriminant segment inside and outside of the RF, based on the decaying response phase (50-250ms after stimulus offset; gray shaded region in Figure 3B) and included the three shortest stimulus durations of the worth experiment. The SVM defines a hyperplane in a space with one dimension per recording site that uses the decaying neuronal activity to maximally separate trials in which the stimulus had or had not been in the RF. The SVM-score of a particular trial is the distance between the point in space defined by the activity across recording sites and this hyperplane. The average SVM-score was high, on average, if the discriminant segment fell in the RF and low if it did not. We sorted trials in the condition with the discriminant segment in the RF into those with a high and low SVM-score ($Score_{In}$ in Figure 3D; median split) and compared the monkeys' accuracy between these two groups of trials. We used a bootstrapping method to determine the significance of accuracy differences, resampling trials with the stimulus in the RF (with replacement) and determining the difference in accuracy based on the median split in the resampled data, for a total of 1,000 iterations.

Likelihood ratio test for performance on decay paradigm

We fit the relationship between MOL and performance with a logistic function using the Palamedes toolbox in MATLAB[68]. To assess whether MOL had a significant effect on performance we compared the fits obtained from logistic functions in which the threshold and slope could vary to a restricted model in which the slope was set to zero. The p value was obtained using a likelihood ratio test:

$$LR = -2(\lambda_{restricted} - \lambda_{full})$$

Where $\lambda_{restricted}$ was the log-likelihood of the data under the restricted model and λ_{full} was the log-likelihood under the full model. The value of LR was compared to a χ^2 distribution with 1 degree of freedom to calculate the p value.

Connectedness modulation

To measure the connectedness modulation (ConnMod, Figure 7), we subtracted the response elicited by the non-connected branch (dashed curves in Figures 7C–7E) from that elicited by the connected branch (continuous curves in Figures 7C–7E) in a time-window of 200ms window starting 100ms after the onset of the discriminant segment (pre-cue condition) or the onset of the cue (post-cue conditions). ConnMod is expressed as fraction of the magnitude of the peak response (the normalization was described in the section Electrophysiology, above). A ConnMod value of 0.1, for example, has an amplitude that equals 10% of the peak response. We performed a paired t test across recording sites to assess the significance of the ConnMod during the presentation of the discriminant

segments. We calculated ConnMod for every recording site and for each of the five conditions. We determined the influence of the timing of the central cue by subtracting the ConnMod evoked by the non-cued pairs from that evoked by the cued pair resulting in ΔConnMod. To estimate behavioral d-prime we used the mean value of ΔConnMod in the same window as described above (purple area in Figures 7C–7E), scaled linearly to the behavioral d-prime using linear regression.

Current Biology, Volume *31*

Supplemental Information

A neuronal basis of iconic memory

in macaque primary visual cortex

Rob R.M. Teeuwen, Catherine Wacongne, Ulf H. Schnabel, Matthew W. Self, and Pieter R. Roelfsema

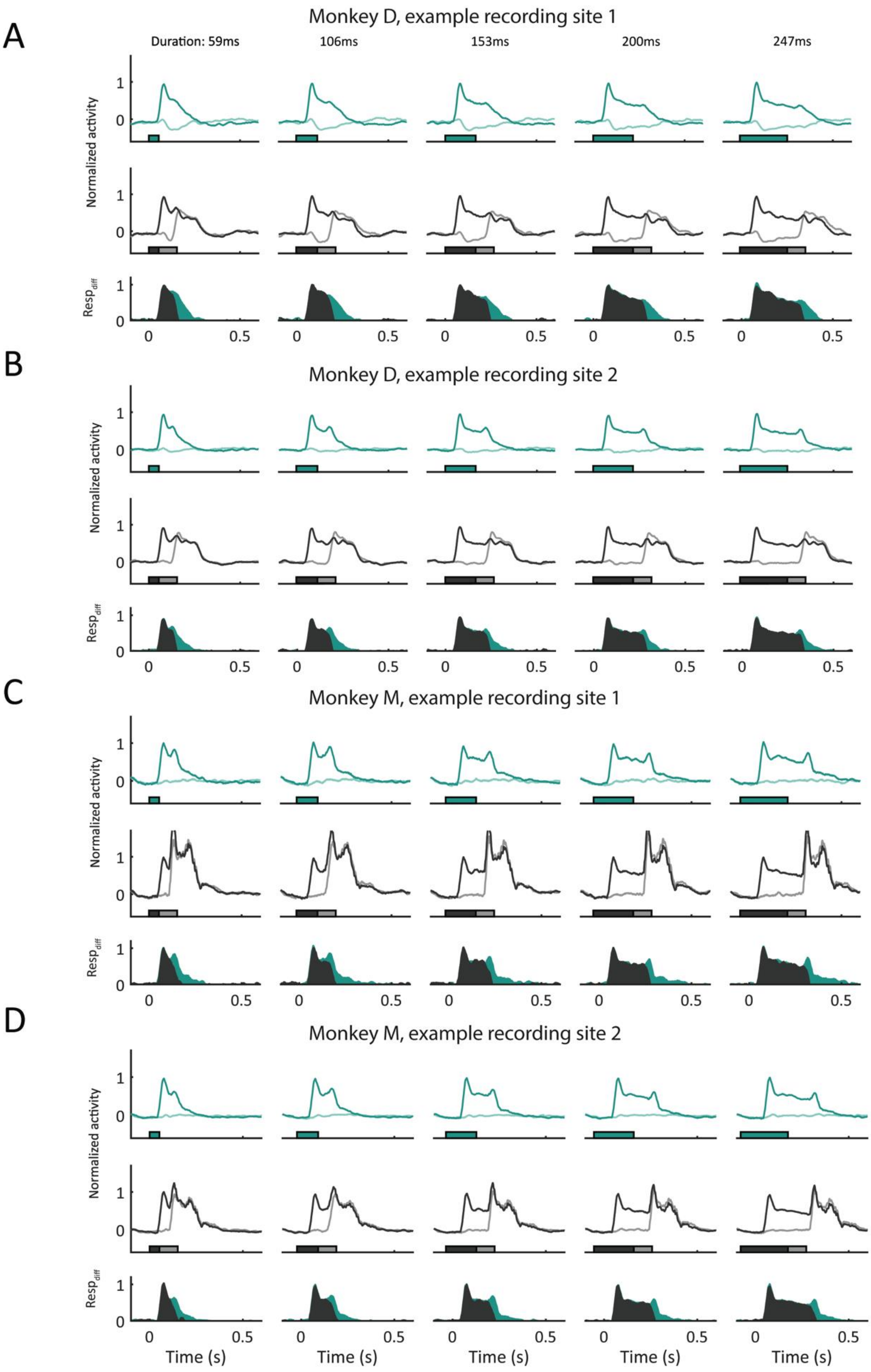
A
Monkey D, example recording site 1
Duration: 59ms
106ms
153ms
200ms
247ms
Normalized activity
Resp$_{diff}$
B
Monkey D, example recording site 2
C
Monkey M, example recording site 1
D
Monkey M, example recording site 2
Time (s)

Figure S1. The neuronal worth at example V1 recording sites. Related to Figure 3. (A,B) Representative V1 recording sites in the worth experiment in monkey D. Dark green and black lines, activity elicited by the stimulus with the discriminant segment in the RF. Light green and grey lines, activity elicited by the stimulus with the discriminant segment outside the RF. In some trials we presented a mask (black, grey) and in other trials the mask was absent (dark and light green). Bars on the x-axis indicate stimulus and mask duration. The panels below represent $Resp_{diff}$, which is the difference in activity elicited by the conditions with the discriminant segment inside and outside the RF in the presence (grey area) and absence of masking (green area). The extra activity difference that occurs without masking is shown as a green area and corresponds to our measure of the neuronal worth. (C,D) Representative recording sites in monkey M.

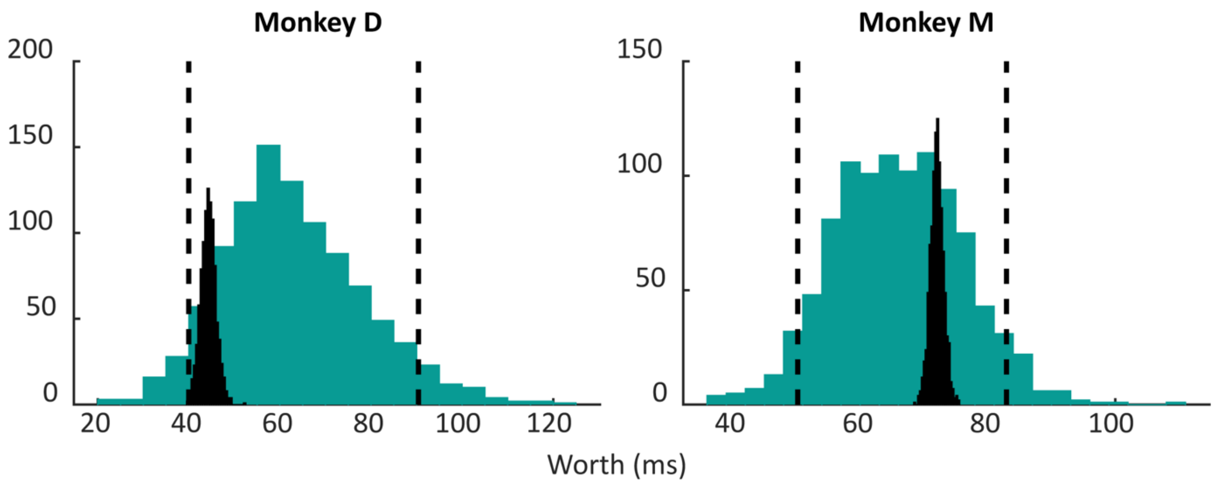

Figure S2. Confidence intervals of the behavioral and neuronal worth. Related to Figure 3. The distributions obtained with bootstrapping for our estimates of behavioral worth (green) and neuronal worth (black). The dashed lines represent the 95% confidence interval of the behavioral worth. The distributions are overlapping, which indicates that the neuronal worth is compatible with the behavioral worth.

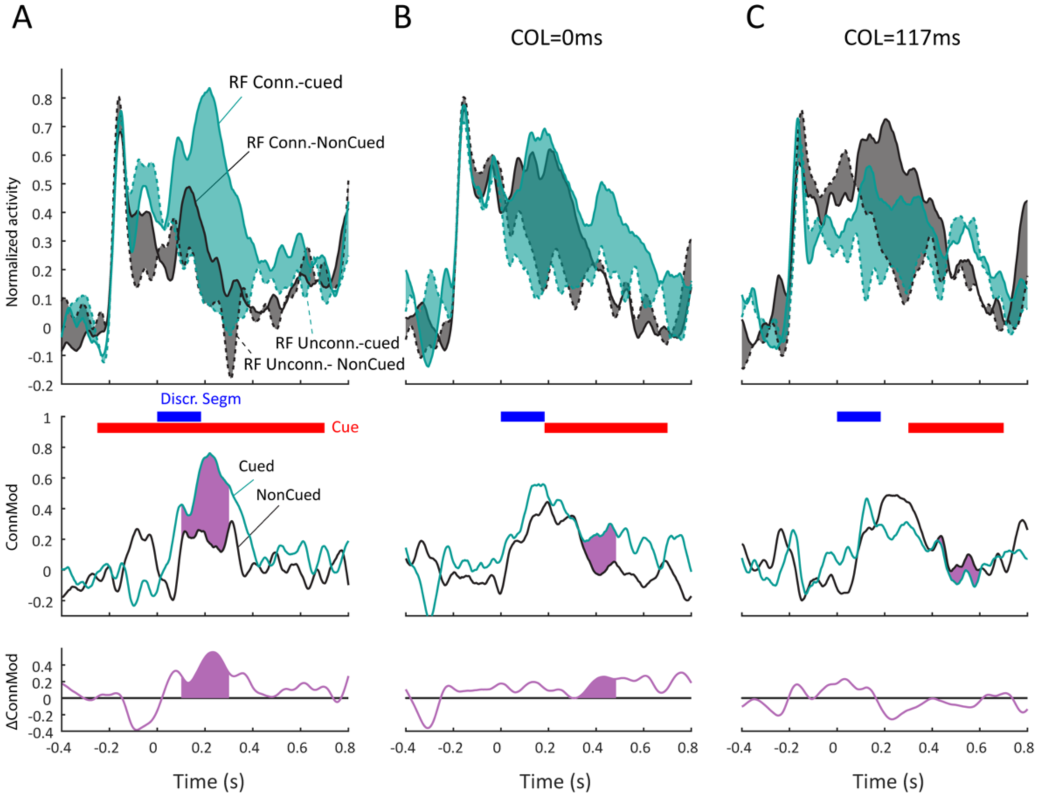

Figure S3. Interactions between iconic memory and the central cue in monkey D. Related to Figure 7. (A-C) V1 activity in the pre-cue condition (A), COL=0 condition (B), and COL=117ms (C), compare to Figure 7C-E (data of monkey M). The contour element in the RF was either connected to the discriminant segment (continuous traces) or not (dashed traces). The pair of curves covering the RF could be cued (green) or not (black). Responses were stronger if the contour in the RF was connected to the discriminant segment than if it was not. ConnMod is the extra activity (green and grey areas) elicited if the RF-contour is connected. The middle panels show the time-course of the connectedness modulation for the cued and non-cued pair of curves, and ΔConnMod (purple area). The lower panels show the time-course of the cueing effect.

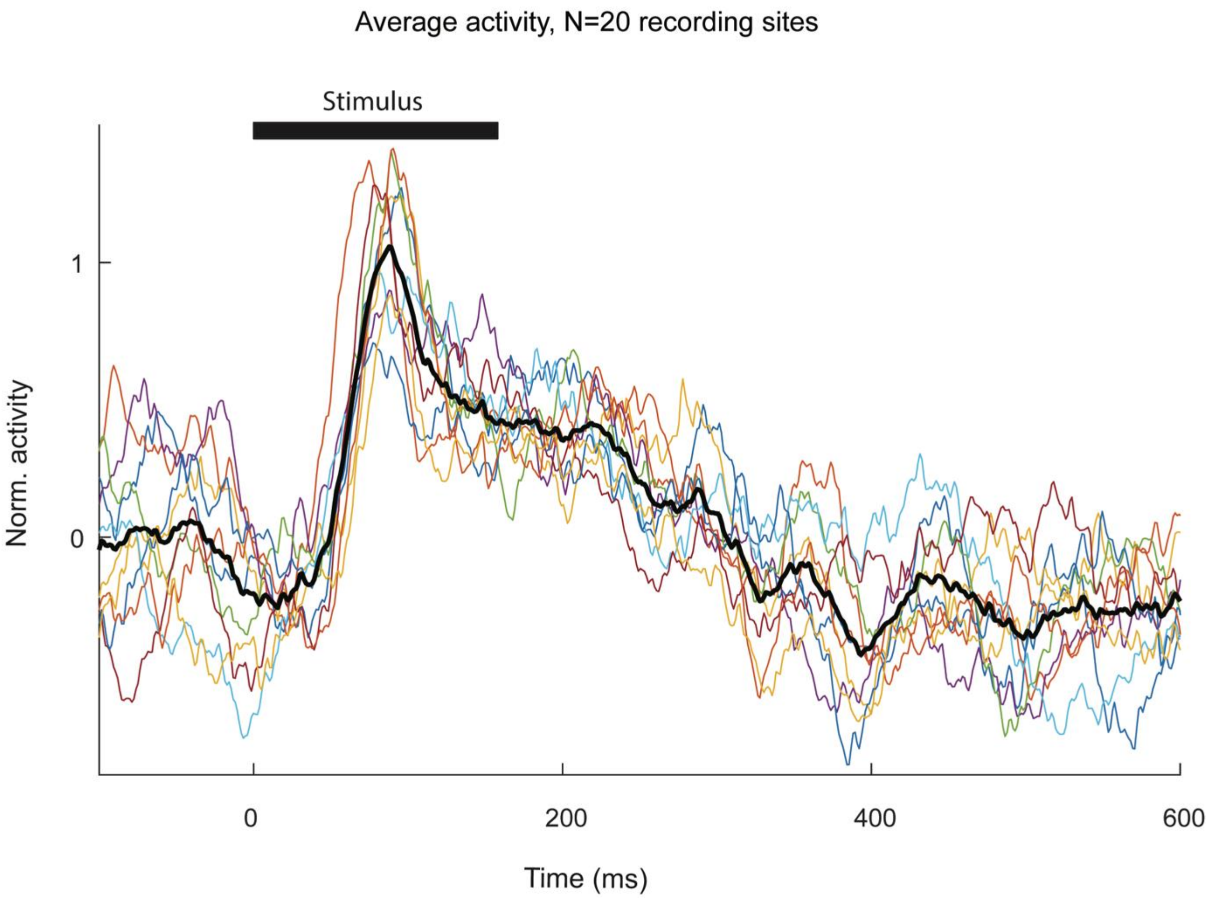

Figure S4. Activity on single trials. Related to Figure 5. Average V1 activity on a number of successive trials averaged across 20 recording sites in monkey D (stimulus duration 158ms, no mask). Colored lines, individual trials. Black line, average across trials in this condition.
